# Molecular epidemiology, evolution, and transmission dynamics of raccoon rabies virus in Connecticut

**DOI:** 10.1093/ve/veae114

**Published:** 2024-12-24

**Authors:** Gabriella Veytsel, Julia Desiato, Hyunjung Chung, Swan Tan, Guillermo R Risatti, Zeinab H Helal, Sungmin Jang, Dong-Hun Lee, Justin Bahl

**Affiliations:** Institute of Bioinformatics, University of Georgia, 120 Green St., Athens, GA 30602, United States; Center for the Ecology of Infectious Diseases, University of Georgia, 140 E. Green Street, Athens, GA 30602, United States; Department of Pathobiology and Veterinary Science, College of Agriculture, Health and Natural Resources, University of Connecticut, 61 North Eagleville Road, Storrs, CT 06269, United States; Connecticut Emerging Infections Program, Yale School of Public Health, 1 Church Street, New Haven, CT 06510, United States; Department of Pathobiology and Veterinary Science, College of Agriculture, Health and Natural Resources, University of Connecticut, 61 North Eagleville Road, Storrs, CT 06269, United States; Department of Infectious Diseases, College of Veterinary Medicine, University of Georgia, 501 D. W. Brooks Drive, Athens, GA 30602, United States; Department of Infectious Diseases, College of Veterinary Medicine, University of Georgia, 501 D. W. Brooks Drive, Athens, GA 30602, United States; Department of Pathobiology and Veterinary Science, College of Agriculture, Health and Natural Resources, University of Connecticut, 61 North Eagleville Road, Storrs, CT 06269, United States; Department of Pathobiology and Veterinary Science, College of Agriculture, Health and Natural Resources, University of Connecticut, 61 North Eagleville Road, Storrs, CT 06269, United States; Department of Geography, Sustainability, Community, and Urban Studies, University of Connecticut, 215 Glenbrook Road, Storrs, CT 06269, United States; Department of Pathobiology and Veterinary Science, College of Agriculture, Health and Natural Resources, University of Connecticut, 61 North Eagleville Road, Storrs, CT 06269, United States; College of Veterinary Medicine, Konkuk University, 120 Neungdong-ro, Seoul 05029, Republic of Korea; Institute of Bioinformatics, University of Georgia, 120 Green St., Athens, GA 30602, United States; Center for the Ecology of Infectious Diseases, University of Georgia, 140 E. Green Street, Athens, GA 30602, United States; Department of Infectious Diseases, College of Veterinary Medicine, University of Georgia, 501 D. W. Brooks Drive, Athens, GA 30602, United States; Department of Epidemiology and Biostatistics, College of Public Health, University of Georgia, 101 Buck Road, Athens, GA 30602, United States

**Keywords:** ecological barrier, spatial epidemiology, phylogeography, phylodynamics, wildlife disease, comparative genomics

## Abstract

In North America, raccoon rabies virus (RRV) is a public health concern due to its potential for rapid spread, maintenance in wildlife, and impact on human and domesticated animal health. RRV is an endemic zoonotic pathogen throughout the eastern USA. In 1991, an outbreak of RRV in Fairfield County, Connecticut, spread through the state and eventually throughout the Northeast and into Canada. Factors that contribute to, or curb, RRV transmission should be explored and quantified to guide targeted rabies control efforts, including the size and location of buffer zones of vaccinated animals. However, population dynamics and potential underlying determinants of rabies virus diversity and circulation in Connecticut have not been fully studied. In this study, we aim to (i) investigate RRV source–sink dynamics between Connecticut and surrounding states and provinces, (ii) explore the impact of the Connecticut River as a natural barrier to transmission, and (iii) characterize the genomic diversity and transmission dynamics in Connecticut. Using RRV whole-genome sequences collected from various host species between 1990 and 2020, we performed comparative genetic and Bayesian phylodynamic analyses at multiple spatial scales. We analyzed 71 whole-genome sequences from Connecticut, including 21 recent RRV specimens collected at the Connecticut Veterinary Medical Diagnostic Laboratory that we sequenced for this study. Our analyses revealed evidence of RRV incursions over the US–Canada border, including bidirectional spread between Quebec and Vermont. Additionally, we highlighted the importance of Connecticut and New York in seeding RRV transmission in eastern North America, including two introduction events from New York to Connecticut that resulted in sustained local transmission. While RRV transmission does occur across the Housatonic and Connecticut Rivers, we demonstrated the distinct presence of spatial structuring in the phylogenetic trees and characterized the directionality of RRV migration. The significantly higher mean transition rates from locations east to west of the Connecticut River, compared to west to east, may be leveraged in directing interventions to fortify these natural barriers. Ultimately, the findings of these international, regional, and state analyses can inform targeted control programs, vaccination efforts, and enhanced surveillance at borders of key viral sources and sinks.

## Introduction

Rabies virus is a single-stranded, neurotropic RNA virus belonging to the genus *Lyssavirus* and the family *Rhabdoviridae*. It is transmissible through saliva, most commonly from a bite or scratch of a rabid animal. Rabies is a vaccine-preventable disease; without timely postexposure prophylaxis (PEP), the virus can reach the brain and spinal cord, at which point it is ∼100% fatal. The canine rabies virus variant, maintained by domestic dogs, is responsible for >99% of human rabies deaths worldwide; dog-associated rabies alone is estimated to cause tens of thousands of deaths and an economic burden of $8.6 billion annually (World Health Organization 2024). Key interventions for reducing human rabies cases include animal control programs, vaccination of wildlife and domestic animals, PEP administration, and health education for healthcare providers and the public. Rabies in dogs was eliminated in the USA in the 1970s, following strong national canine rabies control efforts in the early 1940s. Today, ∼60 000 Americans are treated for rabies exposure each year, primarily from interactions with wildlife and unvaccinated domestic animals (Ma et al. 2020). While all mammals can be infected by rabies virus, only some species are essential in virus transmission and maintenance. In addition to variants that are associated with many species of bats, there are seven distinct antigenic rabies virus variants present in the continental USA that are associated with four terrestrial species: skunks (*Mephitis spp*), raccoons (*Procyon lotor*), arctic foxes (*Vulpes lagopus*), and gray foxes (*Urocyon cinereoargenteus*) (Wallace et al. 2014). The extent to which the virus is specifically adapted to these species is often difficult to tell and evidence is limited. Variants primarily spread within a particular host reservoir and, in major small to midsized carnivore species, are distributed in distinct geographic regions (Ma et al. 2020).

The first cases of raccoon rabies virus (RRV) were documented in Florida in 1947 (Kappus et al. 1970, Scatterday et al. 1960). In the late 1970s, the translocation of raccoons from Florida to Virginia seeded a rabies enzootic in the local rabies-naïve raccoon populations. For the next two decades, RRV quickly spread throughout all mid-Atlantic states, up through all the northeastern states, and crossed into Canada (Nettles et al. 1979, Anderson et al. 2014, Wallace et al. 2014). The rapid spread of RRV in this region was estimated to range from 9.5 to 38.4 km/year (Biek et al. 2007); it may be facilitated by the potential of the reservoir host to reach high population densities, especially in urban areas, where they may live in close contact with humans and their domesticated animals (Szanto et al. 2011, Nadin-Davis et al. 2020). To this day, RRV is distributed along the eastern USA, occasionally spilling over into other mammals. In 1991, an epizootic of RRV began in Fairfield County, Connecticut, and, in the following years, spread throughout the entire state (Smith et al. 2002, Connecticut State Department of Health, n.d.).

The US Department of Agriculture Animal and Plant Health Inspection Service leads the national wildlife vaccination program (Anderson et al. 2014, Ma et al. 2020); most oral rabies vaccination efforts target raccoons and have been successful in containing further westward expansion of RRV from the eastern USA (Department of Agriculture Wildlife Services, n.d.). Eastern Canadian provinces, such as Ontario, New Brunswick, and Quebec, have engaged in RRV vaccination and surveillance efforts along the US–Canada border, in response to cross-border incursions from the eastern USA (Rosatte et al. 2009, Nadin-Davis et al. 2020). Additionally, landscape features can provide a natural barrier or corridor to determine infectious disease spread. Geographical structures, such as water bodies and mountain ranges, have been associated with the geographical clustering of raccoon rabies cases (Recuenco et al. 2007, Wheeler and Waller 2008) and play a role in slowing, and even redirecting, the spread of racoon rabies in certain states (Smith et al. 2002, 2005, Russell et al. 2004, Anderson et al. 2014). At 410 miles (660 km), the Connecticut River is the longest river in New England, crossing entirely through Connecticut, Massachusetts, New Hampshire, and Vermont. Understanding and quantifying the impact of such major geographic barriers can support strategies to curb transmission (Wheeler and Waller 2008, Russell et al. 2006).

The population dynamics and potential underlying determinants of RRV circulation in Connecticut have not been fully studied. In general, few phylogenetic studies have been performed on RRV whole-genome sequences (Nadin-Davis et al. 2017, 2018, 2020, Trewby et al. 2017, Brunt et al. 2020, Chung et al. 2022). Several studies used individual genes (Biek et al. 2007, Szanto et al. 2011) that can over- or underestimate substitution rates (Hanke et al. 2016), contain fewer mutations, and offer less phylogenetic resolution (Dudas and Bedford 2019, Holtz et al. 2023). Furthermore, studies have largely focused on a small number of sequences from one geographical area (Nadin-Davis et al. 2017, Brunt et al. 2020, Chung et al. 2022). In this study, we analyzed 71 whole-genome sequences from Connecticut, including 21 recent RRV specimens collected at the Connecticut Veterinary Medical Diagnostic Laboratory (CVMDL) that we sequenced for this study, in the context of the northeastern USA (*n* = 221) and eastern North America (*n* = 304). We aim to (i) investigate the source–sink dynamics of Connecticut in the context of North America, (ii) explore the impact of the Connecticut River as a natural barrier to transmission, and (iii) characterize the genomic diversity and transmission dynamics in Connecticut.

## Materials and methods

### Rabies virus samples

Brain tissue samples from rabies cases submitted between 2017 and 2020 to CVMDL, including 19 samples collected in Connecticut, 1 sample from Massachusetts, and 1 sample from Rhode Island, were used in this study. A total of 21 brain tissue samples from raccoons (*n* = 15), skunks (*n* = 2), a bobcat (*n* = 1), a cow (*n* = 1), a woodchuck (*n* = 1), and a feline (*n* = 1) were confirmed to be rabies-positive at CVMDL using the direct fluorescent antibody test (Meslin et al. 1996) and quantitative reverse transcription PCR assay (Gigante et al. 2018). The protocols for primer selection, viral RNA extraction, and multiplex tiling reverse transcription PCR (RT-PCR) were described previously (Chung et al. 2022). Briefly, viral RNA was extracted from the cerebellum, hippocampus, and brainstem using TRIzol reagent (ThermoFisher Scientific, USA); next, all RRV genome segments were amplified simultaneously by multiplex RT-PCR using the previously published primer sets for RRV (Nadin-Davis et al. 2002, Campos et al. 2011).

### Genome sequencing and assembly

Genome sequencing of purified RT-PCR products was conducted using the Illumina iSeq 100 or MiSeq platform at the Genomics and Molecular Epidemiology Research (GaMER) lab at the University of Connecticut. Prior to analyzing the data, FastQC version 0.11.9 (http://www.bioinformatics.babraham.ac.uk/projects/fastqc/) was used to check the quality of the raw sequencing data. Reads were analyzed using a workflow on the Galaxy platform (Jalili et al. 2020) that utilized Trimmomatic version 0.38.0 (Bolger et al. 2014) to trim primer and Illumina adapter sequences and filter short (<50 bp) and low-quality sequences (<Q30). Bowtie2 assembler version 2.3.4.3 (Langmead and Salzberg 2012) was used to map the reads to a known reference rabies genome sequence from Connecticut (GenBank accession ON986428) with default parameter settings. iVar consensus version 1.2.3 (Castellano et al. 2021) was used to generate consensus sequences. Geneious Prime 10 Software (http://www.geneious.com/) was then used to generate consensus sequences with parameters set as default except for one alteration to change the threshold to 0% majority for visualization and confirmation of consensus sequences. At a threshold of 0%, the most frequent base in each position will be called. We aligned the newly obtained consensus sequences using MAFFT alignment software v.7 (Katoh and Standley 2013).

### Dataset design

We used three different datasets in this study to reconstruct the evolution and transmission of RRV at different scales. To support targeted RRV interventions, we examined borders of potential viral sources and sinks, including state and international boundaries, geographic structures, and host barriers.

#### North America dataset

On 1 August 2024, we downloaded all available *Lyssavirus* rabies complete genomes from multiple hosts in the USA and Canada from the National Center for Biotechnology Information (https://www.ncbi.nlm.nih.gov/) (*n* = 1156), which includes the 21 newly sequenced genomes. Rabies virus variant typing was not available, so the variant was assumed based on the geographic region, an assumption that has been validated by several studies (Wallace et al. 2014). In the USA, data outside of the Northeast region are very sparse and thus were not included. We also included eastern Canada, as incursions of RRV have been reported in three Canadian provinces (Szanto et al. 2011). Upon examination of our preliminary maximum likelihood tree (*n* = 768 sequences), we root our tree at the midpoint in order to filter out the most divergent sequences (Supplementary Figure S1), resulting in a dataset of 556 sequences, including Vermont (*n* = 66), Connecticut (*n* = 71), Maine (*n* = 39), New York (*n* = 212), New Brunswick (*n* = 32), Quebec (*n* = 52), and Ontario (*n* = 84). Preliminary analysis from tip trait randomization revealed evidence of sampling bias toward New York (Supplementary Table S1 and Supplementary Figure S2), and therefore we took a random sample of 50 sequences in New York. Our final dataset consisted of 304 sequences, including 71 focal Connecticut sequences and, in order to lessen computational demand, a random sample of 70% of each state or province in our contextual dataset: Vermont (*n* = 51), Maine (*n* = 26), New York (*n* = 32), New Brunswick (*n* = 24), Quebec (*n* = 38), and Ontario (*n* = 62).

#### Connecticut River dataset

We linked our North American dataset to latitude/longitude data provided by the University of Connecticut and the study by Nadin-Davis et al. (2020). If latitude/longitude was missing, we geocoded city or zip code using the zipcodeR (Rozzi 2021) and tidygeocoder (Cambon et al. 2021) packages in R (R Core Team 2021). We downloaded the USA Rivers and Streams feature layer from ArcGIS Hub (https://hub.arcgis.com/datasets/esri::usa-rivers-and-streams/about) and used rnaturalearth (Massicotte and South 2017), maps (Becker et al. 2003), and sf (Pebesma 2018) packages in R (R Core Team 2021) to plot the rivers, state/county boundaries, and latitude/longitude associated with the sequence data. Given that raccoons have been reported to travel up to 150 miles (241 km) (Pennsylvania Game Commission 2021), we subset the dataset to points that fall within a 150-mile buffer around the Connecticut River (Supplementary Figure S3). After the removal of an outlier (MN418166|Dog|New York), our dataset consisted of 297 sequences, including Connecticut (*n* = 71), Maine (*n* = 28), Vermont (*n* = 72), and New York (*n* = 127). Preliminary analysis from tip trait randomization revealed evidence of sampling bias toward New York (Supplementary Figure S4 and Supplementary Table S2); we therefore took a random sample of 50 sequences in New York, resulting in a final dataset of 221 sequences.

#### Connecticut dataset

We filtered our dataset to include only Connecticut sequences (*n* = 71), including two sequences that were sequenced by CVMDL, which fell just outside the border in Rhode Island and Massachusetts, but clustered genetically with the Connecticut sequences. The dataset includes Litchfield (*n* = 6), Middlesex (*n* = 5), Fairfield (*n* = 16), Hartford (*n* = 7), New Haven, (*n* = 10), New London (*n* = 7), Tolland (*n* = 10), and Windham (*n* = 10) counties. Sequences were collected from raccoons (*n* = 51), skunks (*n* = 9), woodchucks (*n* = 3), feline (*n* = 2), cow (*n* = 2), bobcat (*n* = 2), foxes (*n* = 1), and deer (*n* = 1).

### Raccoon rabies virus case report data

The Connecticut Department of Public Health recorded all rabies cases in the state of Connecticut from 2000 to 2019 in annual line lists based on county and host species. To characterize the spatial distribution of RRV cases in Connecticut, we created an interactive ArcGIS web map that illustrates the distribution of cases reported by county in Connecticut during 1 January 2000 to 31 July 2019 (Lee and Jang, n.d.).

### Pairwise genetic distance

We calculated the amount of genetic variation present in the population by computing the pairwise genetic distances between isolates from an alignment using the *ape* package in R (Paradis and Schliep 2019). We used the dist.dna() function to compute a matrix of pairwise distances based on DNA sequence alignment and calculated the proportion of sites that differed between each pair of sequences as a percentage of sequence dissimilarity. We computed the pairwise genetic distance among and between RRV clades and locations. This allowed us to explore the relationship between genetic diversity and spatial structure.

### Temporal signal and model choice

To examine the relationship between genetic divergence and time, we reconstructed a phylogenetic tree in IQ-TREE v.1.6.12 (Nguyen et al. 2015) without assuming a molecular clock. General time reversible (GTR) + F + I + G4 was selected by ModelFinder (Kalyaanamoorthy et al. 2017) as the best-fit model based on both the Akaike Information Criterion and the Bayesian Information Criterion. In TempEst (Rambaut et al. 2016), we examined the temporal signal of our rabies virus sequence data and looked for problematic sequences. We identified MN418166 as an outlier, as it demonstrated too many unique differences compared to its ancestor per the residual, tree, and root-to-tip plots. To formally confirm the result from TempEst, we conducted a Bayesian Evaluation of Temporal (BETS) analysis to examine the strength of the temporal signal of our dataset and whether a molecular clock can be applied using the sampling dates of these sequences. For both strict and relaxed clock models, we compared the fit to the data of two models: a model in which the data have the actual (heterochronous) sampling times and a model without sampling dates. We used generalized stepping-stone sampling, as it is currently the best approach to estimate the (log) marginal likelihood of models (Baele et al. 2016, Fourment et al. 2020).

### Bayesian ancestral reconstruction

In Bayesian evolutionary analysis by sampling trees (BEAST, v.1.10.4) (Suchard et al. 2018), coalescent analysis was performed by 3–5 independent runs of 100–150 million Markov chain Monte Carlo (MCMC) iterations, with 10% burn-in, using the GTR substitution model with empirical base frequencies and Gamma-distributed rate variation among sites with four discrete categories. The mean rates for both the strict and relaxed molecular clock models were specified under a lognormal distribution with a mean value of 2.5 × 10^−4^, based on the literature (Nadin-Davis et al. 2017), and a standard deviation of 1 × 10^−4^. Similarly, the prior for the time to the most recent ancestor (tMRCA) was specified under a normal distribution, with a mean of 1985 and a wide standard deviation of 15. We applied a Skygrid prior to capture complex population dynamics. We confirmed adequate mixing and convergence by examining parameter traces and effective sample size values using Tracer v1.7.1 (Rambaut et al. 2018). We combined tree files from 3 to 5 independent runs in LogCombiner v1.10.4 with 10% burn-in and resampled them at a frequency to generate 9–10 000 trees. These were summarized in TreeAnnotator v1.10.4 (Suchard et al. 2018) onto a single time-scaled maximum clade credibility (MCC) tree and visualized in R version 4.4.0 (R Core Team 2021) using the ggtree package (Yu et al. 2017).

### Discrete trait analysis

We used an ancestral discrete trait reconstruction approach to understand patterns of spatial diffusion and cross-species (raccoon, skunk, and other species) transmission dynamics of RRV. In Connecticut, samples were collected from all eight counties. In the Connecticut River analyses, as the Connecticut River intersects Hartford and Middlesex counties, we divided them into east and west portions by latitude/longitude. All discrete trait analyses were performed on a set of empirical starting trees estimated under a strict molecular clock model, an asymmetric substitution model, and a Bayesian Stochastic Search Variable Selection (BSSVS) framework. The BSSVS procedure limits the number of rates to only those that adequately explain the phylogenetic diffusion process. For the phylogeographic analyses, we estimated Markov jump counts to quantify the number of inferred location state transitions along phylogenetic branches modeled by a continuous-time Markov chain (CTMC) process. Additionally, we estimated Markov rewards to measure the duration of time viruses spend in each location state as a proportion of the time represented by the tree. Mean transition rates and mean rewards were summarized in Tracer; SPREAD4 (Nahata et al. 2022) was used to summarize Bayes factor (BF) values and posterior probabilities (PPs) for the most likely transitions; Markov jumps were extracted using a Perl script (BEAST Community 2017); and statistical analyses were performed using R Statistical Software version 4.4.0 (R Core Team 2021).

### Generalized linear model

We performed an additional discrete trait analysis, in which we parameterized the CTMC matrix as a generalized linear model (GLM) to identify predictors for spatial diffusion. We used the orientation to the Connecticut River as our binary predictor, with 1 s to indicate that sequences are on the same side of the river (east or west) and 0 s if they are not. We considered sequence sample sizes, which can bias ancestral reconstructions, for both the “donor” and “recipient” as additional predictors.

### Sampling bias

Ancestral reconstruction may be biased by the number of sequences sampled from each location. To test whether our inferences were sensitive to sampling heterogeneity, we performed an additional analysis in which we randomized the location assignments at the tips throughout the MCMC. We did this by adding a new MCMC transition kernel (i.e. operator) that swaps location states between two randomly selected taxa (Edwards et al. 2011). Sensitivity analysis was performed for each discrete trait analysis in which sequence traits (location) were randomly switched throughout the Bayesian analysis. The ancestral root state probabilities (i.e. the probability that the most recent ancestor existed in a given location) were used to assess the influence of sampling bias in several ways. First, we expect the ancestral state probability at the root of the tree to be the same for all states. Second, we investigated whether there was a correlation between location sampling frequency and root state probability, measured by the Pearson correlation coefficient (*R*), suggesting that our estimates are informed by the sampling scheme, rather than spatial structure in our data. Third, we compared the ancestral state probability pre- and postrandomization.

## Results

### Temporal signal and model choice

BETS analyses for all three datasets formally confirmed the root-to-tip regression results from TempEst (Supplementary Figure S5). The strongly positive (log) BFs indicate that these RRV data exhibit a clear temporal signal, which is critical for generating reliable inferences in BEAST. In the North America dataset, log (BF) values for the relaxed and strict clock models were 322.00 and 487.35, respectively; (log) BF of 5.01 was calculated in favor of the relaxed clock over the strict clock model (Supplementary Table S3). In the Connecticut River dataset, log (BF) values for the relaxed and strict clock models were 233.47 and 490.92, respectively; (log) BF of 21.09 was calculated in favor of the relaxed clock over the strict clock model (Supplementary Table S4). In the Connecticut dataset, (log) BF values for the relaxed and strict clock models were 29.39 and 26.75, respectively; (log) BF of 8.44 was calculated in favor of the strict clock over the relaxed clock model (Supplementary Table S5).

### North America

**Figure 1.**
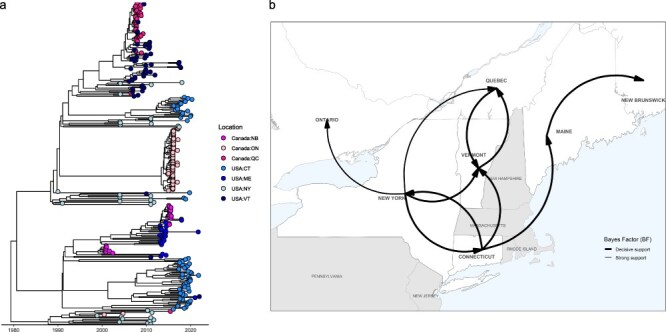
(a) Phylogeny of 304 sequences from eastern North America. Sequences were collected from New Brunswick, Ontario, Quebec, Connecticut, Maine, New York, and Vermont. (b) Map showing statistically supported transitions by location. Line thickness corresponds to BF values, ranging from strong to decisive support.

To track the diffusion of RRV in the Northeast, including into and out of Connecticut, we investigated source–sink dynamics. Three main clades were identified from the phylogeny of 304 North American sequences. The common ancestor was inferred as New York (PP = 0.99), dating back to 29 March 1979 [95% highest posterior density (HPD): 1 November 1975, 20 August 1982] (Fig. 1). The first clade contained sequences from Ontario (*n* = 60), Quebec (*n* = 37), Connecticut (*n* = 24), New York (*n* = 21), and Vermont (*n* = 49). Connecticut sequences clustered closely with sequences collected from New York. A total of 60 Ontario sequences appeared on a long branch and were tightly clustered, with an estimated most recent common ancestor (MRCA) of April 2013, capturing the Hamilton RRV outbreak that resulted from an incursion from eastern New York around 2013 (Nadin-Davis et al. 2020). Unlike Clades 1 and 3, which have a common ancestor from New York (PP = 1.0 and 0.9998, respectively), Clade 2 has a common ancestor from Connecticut (PP = 0.9979). This clade contained sequences from New Brunswick (*n* = 24), Connecticut (*n* = 47), Maine (*n* = 26), and Vermont (*n* = 2). Here, the Connecticut sequences clustered tightly with a few sequences from Maine and Vermont. The third clade is composed of 11 New York sequences, 2 Ontario sequences, and 1 Quebec sequence. Within these major clades, subclades are mostly geographically constrained, with limited transmission from surrounding states; the exceptions include transmission between Quebec and Vermont, Maine and New Brunswick, and New York, Ontario, and Quebec, potentially indicating transboundary outbreaks. Of note, the phylogeny reveals four introductions from New York into Connecticut, but only two that result in sustained transmission.

**Figure 2.**
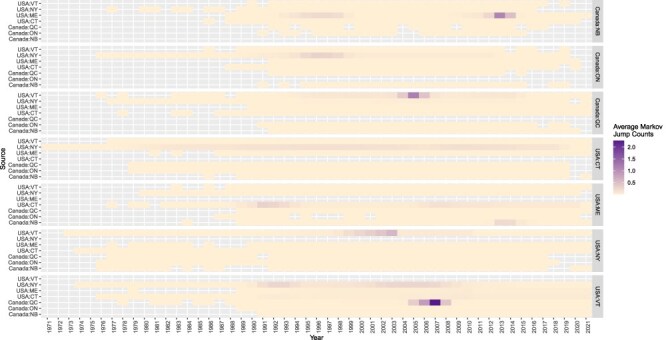
Average Markov jump counts over time, North America. Upon examination of the average Markov jump counts over time, it appears that New York is a relatively low, but consistent source of RRV into Connecticut and Vermont during at least half of the study period. On the other hand, several sources exhibit spikes of RRV transmission during a short time interval, including Quebec to Vermont, Vermont to Quebec, and Maine to New Brunswick, which may suggest potential outbreaks.

BEAST analysis revealed a molecular clock rate of 2.4 × 10^−4^ nucleotide substitutions per site across the genome for these sequences (95% HPD: 2.3 × 10^−4^, 2.6 × 10^−4^). Of the 42 transition rates, the results of our discrete trait analysis revealed the following statistically supported migration rates: Connecticut to Maine (0.86 transitions/year), New York (0.02 transitions/year), and Vermont (0.46 transitions/year); Maine to New Brunswick (1.36 transitions/year); New York to Connecticut (1.42 transitions/year), Vermont (1.05 transitions/year), Ontario (0.71 transitions/year), and Quebec (0.18 transitions/year); and Vermont to Quebec (1.48 transitions/year) and Quebec to Vermont (2.23 transitions/year) (Fig. 1 and Table 1). Markov jump counts estimated that the highest average number of transitions occurred from New York to Connecticut (*n* = 5.09), Quebec to Vermont (*n* = 4.03), New York to Vermont (*n* = 3.62), Vermont to Quebec (*n* = 3.48), and Maine to New Brunswick (*n* = 3.06) (Supplementary Figure S6). Upon examination of the average Markov jump counts over time, it appears that New York is a relatively low, but consistent source of RRV into Connecticut and Vermont during at least half of the study period (Fig. 2). On the other hand, several sources exhibit spikes of RRV transmission during a short time interval, including Quebec to Vermont, Vermont to Quebec, and Maine to New Brunswick, which may suggest potential outbreaks. As a proportion of time represented by the tree, Markov rewards estimated that viruses circulated the longest in New York (32.0%), Connecticut (30.9%), Vermont (14.2%), and Maine (1.3%), while the least time in Ontario (5.5%), Quebec (3.0%), and New Brunswick (1.7%). These Markov reward estimates demonstrate the importance of both Connecticut and New York in seeding infections of RRV in eastern North America.

**Table 1.** Mean transition rates between locations, North America.

		Sink						
		USA: CT	USA: ME	USA: NY	USA: VT	Canada: NB	Canada: ON	Canada: QC
Source	USA: CT	–	**0.86 (0, 1.89)** †	**0.02 (0, 0.02)** †	**0.46 (0, 1.52)** †	0.02	0.01	0.03
	USA: ME	0.03	–	0.03	0.45	**1.36 (0.14, 2.95)** †	0.02	0.04
	USA: NY	**1.42 (0.27, 2.85)** †	0.01	–	**1.05 (0, 2.44)** †	0.01	**0.71 (0, 1.63)** *	**0.18 (0, 0.96)** *
	USA: VT	0.05	0.03	1.09	–	0.03	0.03	**1.48 (0.16, 3.20)** †
	Canada: NB	0.06	0.48	0.05	0.08	–	0.06	0.06
	Canada: ON	0.08	0.07	0.08	0.08	0.07	–	0.09
	Canada: QC	0.05	0.05	0.05	**2.23 (0.31, 4.70)** †	0.05	0.04	–

BSSVS statistically supported transition rates with 95% Bayesian credible intervals, where the PP > 50% and BF > 16, are given in bold.

* BF 11–100 (strong support).

† BF >100 (decisive support).

### Connecticut River

**Figure 3.**
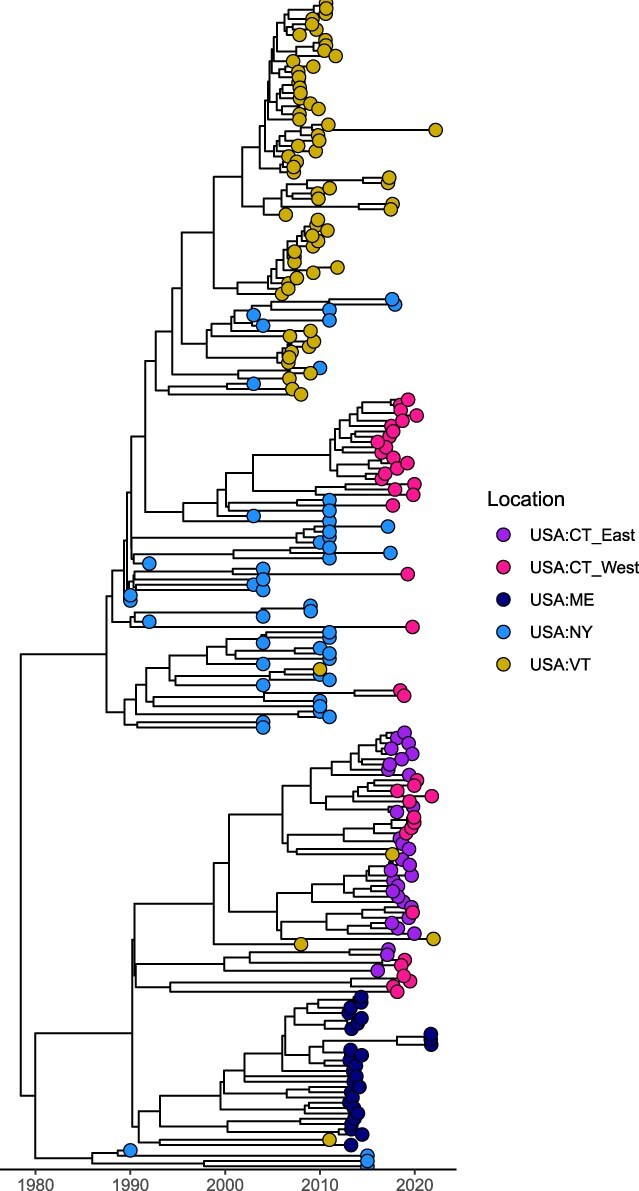
Phylogeny of 221 sequences from the Northeast. A time-scaled MCC tree was generated using 221 sequenced RRV samples from Maine, New York, Vermont, and eastern and western Connecticut. All sequences east of the Connecticut River (eastern Connecticut and Maine) fall into the second main clade. While the first clade is composed entirely of sequences west of the Connecticut River (New York, Vermont, and western Connecticut), the second clade demonstrates transmission between east and west locations. The third main clade is just four sequences from New York.

To investigate the impact of the Connecticut River on RRV transmission, we analyzed the diversification of sequences across geographic locations. The resulting phylogeny of 221 sequences identified three main clades, with an ancestor in New York (PP = 0.98) dating back to 20 April 1978 (95% HPD: 6 July 1974, 6 July 1981) (Fig. 3). We observed diversification between eastern Connecticut sequences, which are only found in the second clade, and western Connecticut sequences, which fall into Clades 1 and 2. The first main clade is composed of sequences from western Connecticut (*n* = 24), New York (*n* = 46), and Vermont (*n* = 68). The second clade is made up of eastern Connecticut (*n* = 31), Western Connecticut (*n* = 16), Maine (*n* = 28), and Vermont (*n* = 4). We observed spatial structure in the phylogeny, since Clade 1 is made up entirely of sequences west of the Connecticut River (Vermont, New York, and western Connecticut), while all sequences east of the Connecticut River fall into Clade 2. We did not observe complete isolation, however, as there was transmission occurring between locations east and west of the river in Clade 2. Unlike Clades 1 and 3 that have a common ancestor from New York (PP = 1.0, 0.9993, respectively), Clade 2 has a common ancestor from eastern Connecticut (PP = 0.86). The third clade consisted of four sequences from New York.

**Figure 4.**
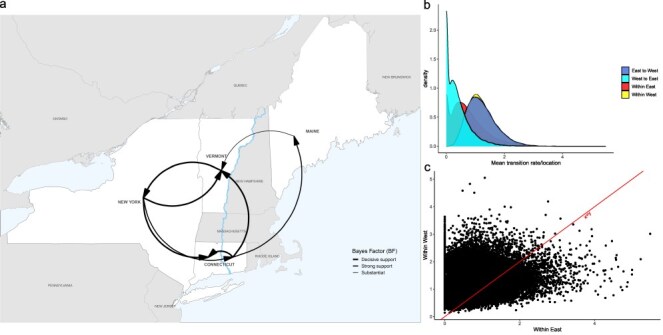
Inferred statistically supported migration rates and patterns, Connecticut River. (a) Map showing statistically supported transitions by location. Line thickness corresponds to BF values, ranging from substantial to decisive support. (b) Density distribution of statistically supported mean transition rates between locations shows that transitions among locations west of the river and between east-to-west locations are significantly higher than transitions west to east or within locations east of the river. (c) Statistically supported mean migration rates per MCMC step of east-to-east transitions versus west-to-west transitions.

We applied two models in our discrete trait analyses: the asymmetric substitution model and the generalized linear model. The asymmetric substitution model provides valuable insights into the patterns of transmission dynamics of the virus across states, while the GLM further investigates key predictors for patterns of spatial spread. Based on a PP ≥ 50% and BF ≥ 3.2 (Kass and Raftery 1995, Bouckaert 2021), we estimated statistical importance of transition rates between locations. Overall, locations west of the river and from east to west of the river had significantly higher transition rates compared to those among locations east of the river and from west to east (Fig. 4). Of the 20 possible transitions, our discrete trait analysis revealed the following eight statistically supported migration rates: New York to Western Connecticut (1.47 transitions/year), Vermont (0.99 transitions/year), and eastern Connecticut (0.38 transitions/year); Vermont to New York (1.24 transitions/year); Eastern Connecticut to Western Connecticut (1.98 transitions/year), Vermont (1.45 transitions/year), and Maine (0.73 transitions/year); and Maine to Vermont (0.29 transitions/year) (Fig. 4 and Supplementary Table S6). Markov jump counts estimated that the highest average number of transitions occurred among locations west of the river and from east to west, particularly from Eastern Connecticut to Western Connecticut (5.36) and from New York to Western Connecticut (5.28) (Supplementary Figure S7 and Supplementary Table S7). As a proportion of time represented by the tree, there is not a significant difference between the time RRV circulated in eastern Connecticut (17.4%) and Western Connecticut (15.9%). While we find important differences in the directionality of viral migration in orientation to the river (among east, east to west, among west, and west to east), the results of our GLM suggest that its function as a boundary (same side or not) is not an important predictor for patterns of spatial spread (Supplementary Table S8).

### Connecticut

**Figure 5.**
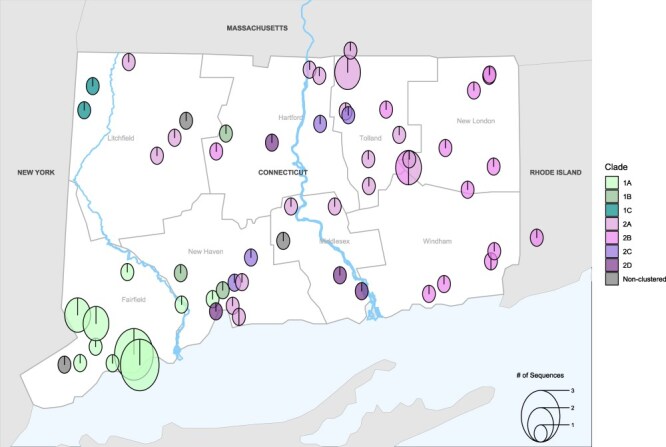
Distribution of RRV variants in Connecticut. The geographical locations of samples analyzed in the phylogeny presented in Figure 1. The size of the circles corresponds to the number of sequences in each location.

We aggregated RRV case report data to determine the distribution of RRV in host species and counties. Between 2000 and 2019, RRV was distributed across 17 different species, predominantly in raccoons (*n* = 1900) and skunks (*n* = 822) (Supplementary Figure S8). Most cases in the state were reported in Fairfield County (*n* = 954), followed by New Haven County (*n* = 674) and Hartford County (*n* = 527) (Lee and Jang, n.d.). Overall, we found that the number of cases in wildlife has remained mostly stable but has decreased since 2015 (Lee and Jang, n.d.). Using sequences that were isolated from 71 of these cases, we visualized the spatial distribution of RRV subclades and hosts in Connecticut. In addition to the Connecticut River, which fully divides Middlesex and Hartford Counties, we found that the Housatonic River fully divides Litchfield County. These two major rivers split the state into three sections (Fig. 5), a grouping we utilized for subsequent phylogenetic and comparative genomic analyses. Section 1 consisted of Clade 1 sequences, Section 3 consisted of Clade 2 sequences, and Section 2 was a mix of Clade 1 and Clade 2 sequences. Gene flow is an important mechanism for genetic variation; given our hypothesis that these rivers serve as a barrier for gene flow between populations, we expect to find more genetic similarity among populations in each section than between them. We calculated the pairwise genetic distance between isolates to visualize the mean proportion of dissimilarity between these three sections. When we examined pairwise genetic distance, we found that isolates were more similar within Section 1 and within Section 3 (mean sequence dissimilarity: 0.5% and 0.69%, respectively) (Supplementary Figure S9), compared to within Section 2 (1.1%), between Section 1 and Section 2 (1.3%), between Section 2 and Section 3 (1.0%), and between Section 1 and Section 3 (1.5%). These results are mirrored upon clade comparison: Clade 1 (0.5%), Clade 2 (0.8%), and between Clade 1 and Clade 2 (1.5%) (Supplementary Figure S10).

**Figure 6.**
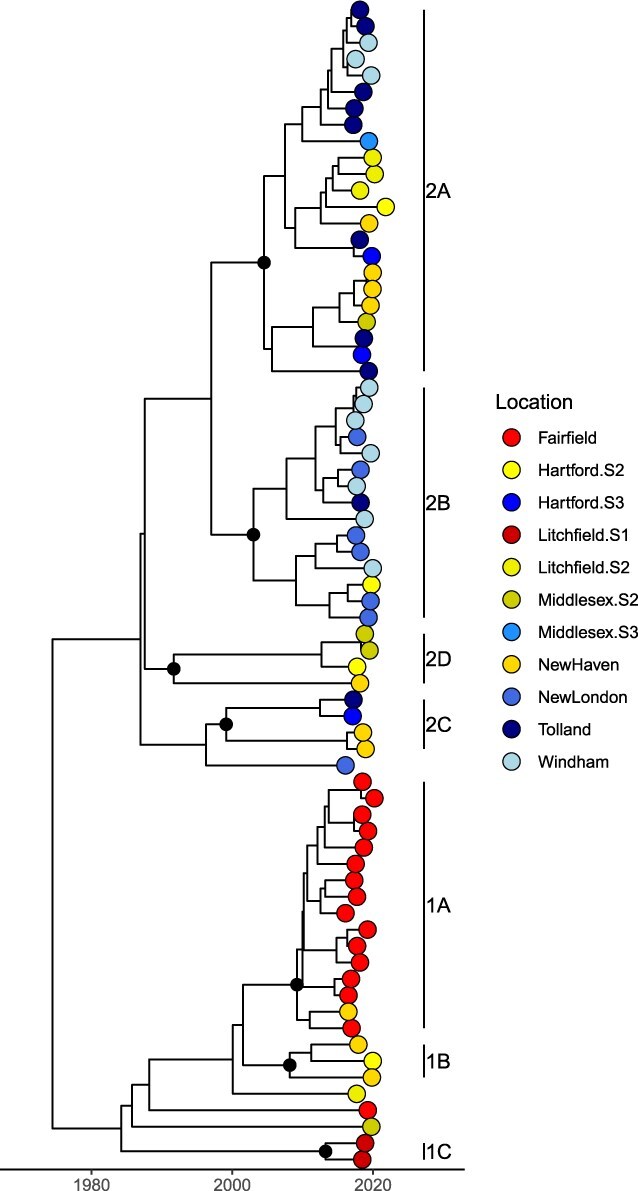
Phylogenetic tree of 71 Connecticut sequences. Clades are labeled, as well as marked with black diamonds at the node.

Using a phylogenetic approach, we sought to characterize RRV and describe spatial diffusion within the state, with county boundaries informed by our Connecticut River analysis. The resulting phylogeny of 71 sequences has an estimated MRCA of 6 June 1974. We identified two main clades (Clade 1 and Clade 2), which we divided into seven subclades (1A, 1B, 1C, 2A, 2B, 2C, and 2D), each with 100% PP (Fig. 6). We estimated the MRCAs of each subclade as Clade 2A: 3 July 2004 (95% HPD: 20 January 1998, 16 July 2009), Clade 2B: 4 November 2002 (95% HPD: 28 July 1995, 26 August 2008), Clade 2C: 17 January 1999 (95% HPD: 18 September 1990, 5 May 2006), Clade 2D: 14 October 1991 (95% HPD: 3 October 1979, 31 January 2001), Clade 1A: 3 June 2009 (95% HPD: 30 August 2005, 4 July 2012), Clade 1B: 3 December 2007 (95% HPD: 5 September 2002, 8 June 2012), and Clade 1C: 8 December 2012 (95% HPD: 20 December 2008, 26 August 2015) (Table 2).

**Table 2.** Estimated time of the most recent common ancestor for seven distinct phylogenetic subclades identified in Connecticut.

Clade	MRCA	95% HPD interval	
1A	3 June 2009	30 August 2005	4 July 2012
1B	3 December 2007	5 September 2002	8 June 2012
1C	8 December 2012	20 December 2008	26 August 2015
2A	3 July 2004	20 January 1998	16 July 2009
2B	4 November 2002	28 July 1995	26 August 2008
2C	17 January 1999	18 September 1990	5 May 2006
2D	14 October 1991	3 October 1979	31 January 2001

**Figure 7.**
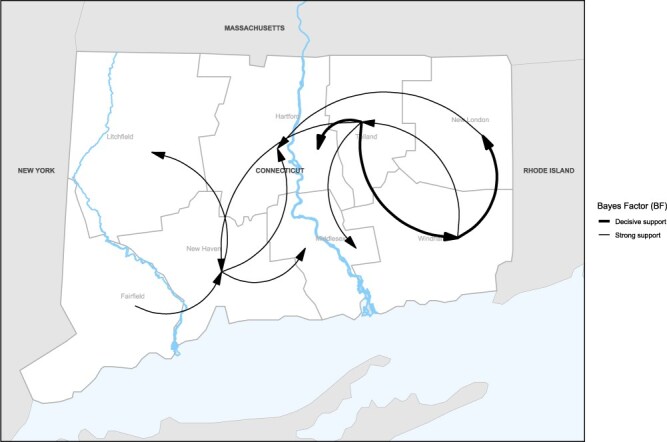
Statistically supported transition rates, Connecticut. Map showing statistically supported transitions by location in Connecticut. Line thickness corresponds to BF values, ranging from substantial to decisive support.

Of the 110 possible transitions, our discrete trait analysis revealed the following 11 statistically supported migration rates: Fairfield to New Haven (0.92 transitions/year); New Haven to Litchfield (Section 2) (0.83 transitions/year), Hartford (Section 2) (1.15 transitions/year), and Middlesex (Section 2) (0.81 transitions/year); Tolland to New Haven (1.33 transitions/year), Hartford (Section 3) (1.18 transitions/year), Middlesex (Section 3) (0.34 transitions/year), and Windham (1.38 transitions/year); New London to Hartford (Section 2) (0.94 transitions/year); and Windham to Tolland (0.99 transitions/year) and New London (2.15 transitions/year) (Fig. 7 and Supplementary Table S9). Ultimately, there are one supported transition rate between Sections 1 and 2 (crossing the Housatonic River) and two supported transition rates between Section 3 and Section 2 (crossing the Connecticut River), compared to three supported transition rates within Section 2 and five supported transitions within Section 3. This suggests that while sequences are not entirely isolated on either side of these rivers, there is a higher proportion of supported rates within these sections than between them. As a proportion of time represented by the tree, Markov rewards estimated that viruses circulated the longest in Tolland (22.1%), New Haven (19.0%), and Fairfield (17.7%) counties (Table 3).

**Table 3.** Mean reward time spent in each location, as the mean sum of branches (per tree), and the proportion of the time represented by the tree.

	Mean sum reward time	95% HPD interval	Proportion (%)
Fairfield	120.99	56.29, 213.01	17.65
Hartford S2	27.90	3.06, 70.57	4.07
Hartford S3	19.34	0.22, 112.37	2.82
Litchfield S1	28.40	4.62, 70.23	4.14
Litchfield S2	31.40	8.69, 70.80	4.58
Middlesex S2	33.90	3.16, 81.75	4.95
Middlesex S3	6.43	0.0008, 17.45	0.94
New Haven	130.40	46.94, 232.13	19.02
New London	44.31	12.67, 105.24	6.46
Tolland	151.29	33.61, 256.49	22.07
Windham	91.79	20.98, 177.60	13.39

We performed an additional discrete trait analysis to explore host transmission dynamics. As expected, we found that the only statistically supported transition rates were from raccoons to skunks and raccoons to other species (Supplementary Figure S11 and Supplementary Table S10). Similarly, we found that viruses spent the most time in raccoons (88.7%), followed by other species (7.4%) and skunks (3.6%). The geographic distribution of host species is mapped in Supplementary Figure S12.

### Sampling bias

While we found evidence of sampling bias toward New York in our preliminary analyses of North America (Supplementary Table S1 and Supplementary Figure S2) and the Connecticut River (Supplementary Table S2 and Supplementary Figure S4), our subsampling schemes corrected for this. We did not find any statistically significant correlation between the number of sequences and the root state probability (*R* = 0.41, *P* = .49 and *R* = 0.32, *P* = .48, respectively). We also found that the ancestral state probabilities were very different pre- and post-tip trait randomization. After subsampling the North America dataset, the root state probability for New York upon tip trait randomization was .4121, compared to the actual probability of .9991 (Supplementary Table S1). After subsampling the Connecticut River dataset, the root state probability for New York upon tip trait randomization was .42, compared to the actual probability of .9848 (Supplementary Table S2). Given the sample size of the Connecticut dataset, we preserved all sequences (*n* = 71). Given 11 states, we expected the root probability for each to be ∼.09. The root state probability converged to the prior expectation where all rates are approximately even (between .03 and .14). Additionally, the ancestral state probability was very different pre- and post-tip trait randomization. The root state probability for Tolland County upon tip trait randomization was .1405, compared to the actual probability of .5257 (Table 4). However, we found a correlation between location sampling frequency and root state probability (*R* = 0.68, *P* = .021), suggesting that our estimates may potentially be informed by the number of taxa in the study (Supplementary Figure S13).

**Table 4.** Root state probabilities pre- and post-tip randomization, Connecticut.

	No. of samples	PP	PP with tip randomization
New Haven	20	0.0356	0.132
Middlesex S3	1	0.0134	0.0318
Middlesex S2	4	0.0278	0.0933
Windham	20	0.1111	0.1328
Hartford S2	4	0.0117	0.085
Tolland	20	0.5257	0.1405
Litchfield S2	4	0.0118	0.081
Litchfield S1	2	0.0674	0.0376
Hartford S3	3	0.0644	0.062
Fairfield	32	0.0838	0.0889
New London	14	0.042	0.1152

## Discussion

RRV poses a significant public health concern in North America, where it has rapidly spread across a large geographical area over the last 60 years. Moreover, there is an ongoing risk of its emergence in new areas or re-emergence by animal importation and wildlife transmission (Holtz et al. 2023). Genomic studies in wildlife can identify incursions into new locations, identify source populations, and enable contact tracing that may support control efforts. Ultimately, understanding the cross-species and spatiotemporal dynamics of rabies is critical for predicting the emergence or spread into new hosts and areas (Smith et al. 2002, Troupin et al. 2016). The success of viral emergence depends on complex and interacting anthropogenic, social, and environmental factors; moreover, studies that explore the dynamics of viral emergence are critical for informing epidemiological decision-making (Hepp 2019, Dellicour et al. 2020, Nahata et al. 2021, Holtz et al. 2023). In this study, we present a comprehensive analysis of key sources of RRV transmission at state and international boundaries, the impact of a natural barrier, and the characterization of RRV genomic diversity and cross-species transmission dynamics in Connecticut.

Phylogeographic analyses have revealed multiple incursions of RRV from the USA to Canada (Biek et al. 2007, Szanto et al. 2011, Nadin-Davis et al. 2017, Trewby et al. 2017); these invasions require extensive and costly responses to prevent the establishment of RRV in native Canadian populations. Although raccoon movements are generally <5 km (Rosatte et al. 2007, 2009, 2010, Cullingham et al. 2008, Dharmarajan et al. 2009, Trewby et al. 2017), long-distance dispersal of raccoons >100 km has been reported; this may be due to deliberate or inadvertent human-mediated translocation (e.g. “hitch-hiking” of infected animals on transport trucks) (Nettles et al. 1979, Bretsky et al. 1997, Rosatte et al. 2009, Trewby et al. 2017). Furthermore, recent studies have highlighted the importance of long-distance translocation events in RRV transmission. For example, Nadin-Davis et al. (2020) found that a long-distance translocation of an infected animal from the New York state was responsible for the largest RRV outbreak in Canada (Nadin-Davis et al. 2020). Additionally, Smith et al. (2002) found that although local transmission accounted for most RRV transmission in Connecticut, long-distance translocation was frequent: 13% of townships reported their first raccoon rabies case in the absence of infections in adjacent townships (Smith et al. 2002). In these cases, interventions may involve providing education and awareness around the risks posed by stowaway wildlife to border officials and personnel involved in transportation of goods.

However, our study does not find evidence of long-distance translocation events. In the phylogenetic tree, we observe relatively geographically constrained clades, with limited transmission to adjacent states and provinces. Statistically supported transition rates from the USA to Canada occur only between bordering states and provinces, including Maine to New Brunswick, New York to Ontario and Quebec, and Vermont to Quebec. The North American phylogeny includes a large clade of mostly Vermont and Quebec sequences that were collected over a short time span, with the most recent common ancestor inferred to be Vermont. This is consistent with the literature, as an incursion of RRV into Quebec, beginning in 2006 and continuing into 2009 when the last case was reported (Rees et al. 2011, Nadin-Davis et al. 2018), was found to be the result of transmission from Vermont (Trewby et al. 2017). Moreover, there was one statistically supported transition rate from Canada to the USA (Quebec to Vermont). These incursions over the US–Canada border, including bidirectional spread, indicate the need for increased vaccination and surveillance efforts along the boundaries of Maine and New Brunswick, Vermont and Quebec, and New York, Quebec, and Ontario to reduce the risk for northern expansion into Canada.

In the Northeast, our analysis revealed that Connecticut is an important source of RRV transmission to Maine, New York, and Vermont, while New York is an important source to Connecticut and Vermont. We observed multiple introductions into Connecticut; however, only two resulted in sustained local transmission. Statistically supported transition rates between Connecticut and Maine and between Connecticut and Vermont represent transmission between nonadjacent states. When we examine the phylogeny, we observe that the two Maine and Vermont sequences are on a longer branch in the Connecticut subclade, potentially indicating missing data from neighboring states (e.g. Massachusetts, Rhode Island, and New Hampshire); therefore, it is likely that missing transmission links, rather than long-distance translocation events, are responsible. Our Connecticut analysis also reveals interesting directionality in gene flow; it appears that, when RRV crossed the Housatonic and Connecticut Rivers, migration was restricted from Section 1 to Section 2 and Section 3 to Section 2, leading to genetic diversification in Section 2 and genetic isolation of Clade 1 and Clade 2 sequences in Section 1 and Section 3, respectively. These findings support directing enhanced surveillance and vaccination efforts along the Housatonic River and the Connecticut River to fortify these natural barriers against RRV spillover into Section 2 of Connecticut.

It is essential to continually monitor cross-species transmission to investigate potential host-switching events, which may impact the evolutionary and ecological patterns of RRV spread. Nadin-Davis et al. (2018) found that while there is strong support for transitions from skunks to raccoons, these switches are rare and may be influenced by sampling bias; they conclude that there is no evidence that skunks act as maintenance hosts for RRV in Vermont (Nadin-Davis et al. 2018). In our study, we found that only transition rates (i.e. cross-species transmission events) from raccoons to skunks and raccoons to other species are statistically supported. As expected, discrete trait analysis also confirmed that raccoons are the origin of all seven subclades in Connecticut and viruses circulated the longest in raccoons, demonstrating their importance in seeding RRV transmission to other hosts.

Geographic barriers like rivers often restrict pathogen transmission (Lucey et al. 2002, Smith et al. 2002, 2005, Biek et al. 2007, Cullingham et al. 2009, Szanto et al. 2011). There is evidence that natural barriers have slowed or prevented further movement of RRV further west (e.g. Appalachian Mountain range) or north into Canada (e.g. Great Lakes and St. Lawrence Seaway) (Szanto et al. 2011). Parameterized with a binary variable to indicate a location’s orientation as either on the same side of the river or not, the results of our GLM do not support its importance as a predictor for spatial spread. While RRV transmission does occur across the Connecticut River, additional analyses provide more detailed dynamics, such as directionality. In this work, we demonstrate the distinct presence of spatial structuring of the phylogeny between sequences east and west of the Connecticut River and characterize the directionality of RRV migration. The spatial structure in the phylogeny indicates reduced gene flow between locations east and west of the Connecticut River. The significantly higher mean transition rates from locations east to west of the river, compared to west to east, may be leveraged in directing enhanced surveillance and vaccination efforts along the Connecticut River to fortify this natural barrier. Smith et al. (2002) used a stochastic spatial model to predict raccoon rabies in Connecticut townships. They found that the Connecticut River slowed the spread of rabies by a factor of 7, responsible for an 11-month delay in the expected appearance of rabies or a 16-month delay without long-distance translocation. This effect of regions with minimal gene flow is similar to a control zone that can be achieved by distributing rabies oral vaccination baits or ground vaccination programs (Smith et al. 2002, Cullingham et al. 2009). Our work builds upon these existing studies, demonstrating that the Connecticut River is a semipermeable barrier for RRV transmission. Thus, natural barriers should be considered in conjunction with strategies designed to control rabies emergence or spread into new areas to create better buffer zones that slow transmission.

There are likely other factors contributing to rabies transmission, which we did not capture in our study, such as highways and mountain ranges. Future studies may benefit from exploring the numerous factors that could affect the likelihood of an animal successfully crossing the river, including its length, width, depth, and velocity and the presence of crossings such as dams, bridges, or ice. The long branches in the Connecticut phylogeny reflect missing data that must have been present in the state but are not currently sequenced and analyzed. Filling this gap would be critical to confirming where the rabies virus originated and how it dispersed in this region. Of note, a statistically supported transition rate from Vermont to New York is inferred in the Connecticut River analysis, which is restricted to eastern New York sequences, but is absent from our North American analysis. Lastly, while we have determined that our results are not sensitive to sampling bias in terms of the number of samples in each location, it is possible that our inferences on New York’s importance as a source for RRV in the Northeast and eastern North America analyses are influenced by the wide temporal range and early collection dates of the New York sequences, compared to other states and provinces.

This study benefits from whole-genome sequences collected partly from large-scale enhanced surveillance programs and regular passive surveillance activities that allow us to generate detailed phylogenetic reconstructions (Nadin-Davis et al. 2020). This work shows that the Connecticut River acts as a semipermeable barrier for RRV spatial diffusion and gene flow. We also provide evidence that Connecticut and New York are important sources for RRV transmission in the Northeast. Our analyses also provide additional evidence for natural barriers as considerations in strategies for rabies virus control programs and support for targeted RRV interventions along borders of New York and Connecticut, Quebec, Ontario, and Vermont; Maine and New Brunswick; and Vermont and Quebec.

## Supplementary Material

veae114_Supp

## Data Availability

Sequences generated through this study have been deposited into GenBank (accession numbers ON986424, ON986427, ON986431, ON986442, ON986456, ON986458-66, ON986470, ON986473, ON986475, ON986478, ON986484, and OR227628-29). Sequence alignments, BEAST XML files, metadata for spatiotemporal analyses, and R code are available in a GitHub repository: https://github.com/Gabriella-Veytsel/RRV.
