# Supplementary material for: Molecular epidemiology, evolution, and transmission dynamics of raccoon rabies virus in Connecticut
Source: Virus Evol. 2024 Dec 24;11(1):veae114. doi: 10.1093/ve/veae114 (PMC11711587; doi:10.1093/ve/veae114)
Supplement: veae114_Supp [file veae114_supp.zip › suppl_data/Veytsel et al 2024 V Evol - Supplementary Tables.docx]

|  | **Before Subsampling** | | | **After Subsampling** | | |
| --- | --- | --- | --- | --- | --- | --- |
|  | **No. Sequences** | **PP with Tip Trait Randomization** | **PP** | **No. Sequences** | **PP with Tip Trait Randomization** | **PP** |
| **USA: CT** | 71 | 0.0005 | 0.0112 | 71 | 0.4633 | 0.0003 |
| **USA: ME** | 39 | 0.0005 | 0 | 26 | 0.0004 | 0 |
| **USA: NY** | 212 | 0.9985 | 0.9888 | 32 | 0.4121 | 0.9991 |
| **USA: VT** | 66 | 0.0001 | 0 | 51 | 0.0005 | 0.0006 |
| **Canada: NB** | 32 | 0.0001 | 0 | 24 | 0.0020 | 0 |
| **Canada: ON** | 84 | 0 | 0 | 62 | 0.0002 | 0 |
| **Canada: QC** | 52 | 0.0003 | 0 | 38 | 0.1216 | 0 |

**S Table 1. Tip trait randomization, North America.** Preliminary analysis from tip trait randomization revealed evidence of sampling bias towards New York (A), we therefore subset New York state to 50 sequences prior to subsampling all contextual sequences to 70% due to computational demand (B) (n=304).

|  | Before Subsampling | | | After Subsampling | | |
| --- | --- | --- | --- | --- | --- | --- |
|  | No. Sequences | PP with Tip Trait Randomization | PP | No. Sequences | PP with Tip Trait Randomization | PP |
| USA: CT (West) | 40 | 0.0134 | 0.0033 | 40 | 0.5255 | 0.0022 |
| USA: NY | 127 | 0.9796 | 0.8943 | 50 | 0.4208 | 0.9848 |
| USA: VT | 72 | 0.0014 | 0.0008 | 72 | 0.0003 | 0.0001 |
| USA: CT (East) | 31 | 0.0053 | 0.1005 | 31 | 0.0071 | 0.0120 |
| USA: ME | 28 | 0.0003 | 0.001 72 | 28 | 0.0463 | 0.0009 |

**S Table 2. Tip trait randomization, Connecticut River, before down-sampling New York state.** Preliminary analysis from tip trait randomization revealed evidence of sampling bias towards New York (A), we therefore subset New York state to 50 sequences (B) (n=221).

|  | Strict clock | Relaxed clock |
| --- | --- | --- |
| No sampling dates | -54211.43 | -54041.07 |
| Sampling dates | -53724.08 | -53719.07 |
| Log Bayes factor | 487.35 | 322.00 |

**S Table 3.** Our BETS analysis for the North America dataset using generalized stepping-stone sampling (GSSS) formally confirms the results from TempEst, with strongly positive (log) Bayes factors demonstrating clear temporal signal in our dataset. Additionally, our (log) marginal likelihood results confirmed our model choice, with a (log) Bayes factor of 5.01 in favor of the relaxed clock over the strict clock model.

|  | Strict clock | Relaxed clock |
| --- | --- | --- |
| No sampling dates | -53099.20 | -52820.66 |
| Sampling dates | -52608.28 | -52587.19 |
| Log Bayes factor | 490.92 | 233.47 |

**S Table 4.** Our BETS analysis for the Connecticut River dataset using generalized stepping-stone sampling (GSSS) formally confirms the results from TempEst, with strongly positive (log) Bayes factors demonstrating clear temporal signal in our dataset. Additionally, our (log) marginal likelihood results confirmed our model choice, with a (log) Bayes factor of 21.09 in favor of the relaxed clock over the strict clock model.

|  | Strict clock | Relaxed clock |
| --- | --- | --- |
| No sampling dates | -29282.468 | -29293.552 |
| Sampling dates | -29255.719 | -29264.16 |
| Log Bayes factor | 26.749 | 29.392 |

**S Table 5.** Our BETS analysis for the Connecticut dataset using generalized stepping-stone sampling (GSSS) formally confirms the results from TempEst, with strongly positive (log) Bayes factors demonstrating temporal signal in our dataset. Additionally, our (log) marginal likelihood results confirms our model choice, with a (log) Bayes factor of 8.44 in favor of the strict clock over the relaxed clock model.

|  |  |  | **Sink** | | | | |
| --- | --- | --- | --- | --- | --- | --- | --- |
|  |  |  | **West** | | | **East** | |
|  |  |  | Connecticut (West) | New York | Vermont | Connecticut (East) | Maine |
| **Source** | **West** | Connecticut (West) | - | 0.09 | 0.08 | 0.22 | 0.12 |
|  |  | New York | **1.47 [0.29, 3.05]***** | - | **0.99 [0.07, 2.31]***** | **0.38 [0, 1.20]**** | 0.04 |
|  |  | Vermont | 0.04 | **1.24 [0, 2.74]***** | - | 0.07 | 0.07 |
|  | **East** | Connecticut (East) | **1.98 [0.40, 3.99]***** | 0.05 | **1.45 [0.20, 3.08]***** | - | **0.73 [0, 1.94]**** |
|  |  | Maine | 0.07 | 0.07 | **0.29 [0, 1.22]*** | 0.14 | - |

* Bayes Factor (BF) Value 3.2-10 (substantial evidence)

**BF 11-100 (Strong support)

*** BF >100 (Decisive support)

**S Table 6. Mean Transition Rates Between States.** Statistically supported transitions are shown in bold and highlighted blue.

| **From** | **To** | **Average** |
| --- | --- | --- |
| **East** | | |
| USA:CT_East | USA:ME | 1.50 |
| USA:ME | USA:CT_East | 0.12 |
| **East to West** | | |
| USA:CT_East | USA:CT_West | 5.36 |
| USA:CT_East | USA:NY | 0.02 |
| USA:CT_East | USA:VT | 3.65 |
| USA:ME | USA:CT_West | 0.02 |
| USA:ME | USA:NY | 0.01 |
| USA:ME | USA:VT | 0.33 |
| **West to East** | | |
| USA:CT_West | USA:CT_East | 0.32 |
| USA:CT_West | USA:ME | 0.11 |
| USA:NY | USA:CT_East | 0.84 |
| USA:NY | USA:ME | 0.05 |
| USA:VT | USA:CT_East | 0.05 |
| USA:VT | USA:ME | 0.06 |
| **West** | | |
| USA:CT_West | USA:NY | 0.01 |
| USA:CT_West | USA:VT | 0.03 |
| USA:NY | USA:CT_West | 5.28 |
| USA:NY | USA:VT | 3.24 |
| USA:VT | USA:CT_West | 0.01 |
| USA:VT | USA:NY | 3.05 |

**S Table 7. Average Markov Jumps, Connecticut River.** The highest average number of transitions occurred from Eastern Connecticut to Western Connecticut (5.36) and from New York to Western Connecticut (5.28).

|  | Coefficient Indicator | Coefficient Parameter |
| --- | --- | --- |
| Sample origin | 0.015 | -0.0004 |
| Sample destination | 0.064 | 0.0268 |
| Connecticut River orientation | 0.047 | 0.0245 |

**S Table 8.** **GLM results**. All predictors are associated with very low inclusion probabilities. The results of our GLM model suggest that orientation to the Connecticut River is not an important predictor for patterns of spatial spread.

|  | | | **Sink** | | | | | | | | | | |
| --- | --- | --- | --- | --- | --- | --- | --- | --- | --- | --- | --- | --- | --- |
|  | | | **Section 1** | | **Section 2** | | | | **Section 3** | | | | |
|  |  |  | Litchfield S1 | Fairfield | Litchfield S2 | New Haven | Hartford S2 | Middlesex S2 | Hartford S3 | Middlesex S3 | Tolland | New London | Windham |
| **Source** | **Section 1** | Litchfield S1 | - | 0.17 | 0.06 | 0.08 | 0.06 | 0.08 | 0.06 | 0.08 | 0.10 | 0.06 | 0.06 |
|  |  | Fairfield | 0.09 | - | 0.08 | **0.92 [0, 2.35]**** | 0.03 | 0.04 | 0.03 | 0.02 | 0.06 | 0.02 | 0.02 |
|  | **Section 2** | Litchfield S2 | 0.16 | 0.20 | - | 0.15 | 0.15 | 0.10 | 0.05 | 0.06 | 0.06 | 0.05 | 0.05 |
|  |  | New Haven | 0.11 | 0.37 | **0.83 [0, 2.17]**** | - | **1.15 [0, 2.78]**** | **0.81 [0, 2.43]**** | 0.02 | 0.06 | 0.10 | 0.04 | 0.03 |
|  |  | Hartford S2 | 0.18 | 0.19 | 0.13 | 0.15 | - | 0.63 | 0.05 | 0.06 | 0.08 | 0.05 | 0.06 |
|  |  | Middlesex S2 | 0.22 | 0.21 | 0.08 | 0.14 | 0.34 | - | 0.05 | 0.07 | 0.08 | 0.06 | 0.06 |
|  | **Section 3** | Hartford S3 | 0.13 | 0.13 | 0.08 | 0.26 | 0.08 | 0.11 | - | 0.30 | 0.33 | 0.08 | 0.89 |
|  |  | Middlesex S3 | 0.14 | 0.14 | 0.09 | 0.11 | 0.09 | 0.10 | 0.08 | - | 0.11 | 0.09 | 0.09 |
|  |  | Tolland | 0.04 | 0.05 | 0.07 | **1.33 [0, 3.16]**** | 0.05 | 0.12 | **1.18 [0, 2.61]***** | **0.34 [0, 1.28]**** | - | 0.06 | **1.38 [0, 3.12]***** |
|  |  | New London | 0.05 | 0.06 | 0.03 | 0.12 | **0.94 [0, 2.46]**** | 0.06 | 0.03 |  | 0.19 | - | 0.20 |
|  |  | Windham | 0.04 | 0.04 | 0.02 | 0.09 | 0.05 | 0.03 | 0.03 | 0.01 | **0.99 [0, 2.84]**** | **2.15 [0, 4.24]***** | - |

BSSVS statistically supported transition rates with 95% Bayesian credible intervals (BCI), where the PP > 50% and BF > 3.2, are bolded and shaded.

Bayes Factor (BF) Values

| * | 3.2-10 | Substantial evidence |
| --- | --- | --- |
| ** | 11-100 | Strong support |
| *** | >100 | Decisive support |

**S Table 9. Mean Transition Rates Between States.** Statistically supported transitions are shown in bold and highlighted blue.

|  | **Raccoon** | **Skunk** | **Other** |
| --- | --- | --- | --- |
| **Raccoon** | - | **1.51 [0.18, 3.30]***** | **1.64 [0.21, 3.58]***** |
| **Skunk** | 0.36 | - | 0.39 |
| **Other** | 0.31 | 0.24 | - |

BSSVS statistically supported transition rates with 95% Bayesian credible intervals (BCI), where the PP > 50% and BF > 3.2, are bolded and shaded.

Bayes Factor (BF) Values

| * | 3.2-10 | Substantial evidence |
| --- | --- | --- |
| ** | 11-100 | Strong support |
| *** | >100 | Decisive support |

**S Table 10. Mean Transition Rates Between Host species.** Statistically supported transitions are shown in bold and highlighted blue. Other is comprised of species with < 3 sequences, including woodchuck, fox, cow, bobcat, deer, and feline.

| Accession | Host | Location | Collection Date |
| --- | --- | --- | --- |
| PP447329 | Raccoon | USA:CT - Hartford | 10/18/2021 |
| OR227628 | Raccoon | USA:CT - Tolland | 8/15/2018 |
| OR227629 | Raccoon | USA:CT - Windham | 12/16/2019 |
| ON986424 | Bobcat | USA:CT - Hartford | 10/11/2019 |
| ON986425 | Bobcat | USA:CT - Hartford | 12/16/2019 |
| ON986426 | Cow | USA:CT - Middlesex | 10/5/2019 |
| ON986427 | Cow | USA:CT - Litchfield | 3/26/2020 |
| ON986428 | Deer | USA:CT - Litchfield | 9/7/2017 |
| ON986429 | Feline | USA:CT - Hartford | 6/5/2018 |
| ON986430 | Feline | USA:CT - Windham | 10/22/2018 |
| ON986432 | Fox | USA:CT - NewHaven | 12/11/2018 |
| ON986433 | Raccoon | USA:CT - Tolland | 3/13/2017 |
| ON986434 | Raccoon | USA:CT - Tolland | 3/21/2017 |
| ON986435 | Raccoon | USA:CT - Tolland | 5/9/2017 |
| ON986436 | Raccoon | USA:CT - Windham | 6/26/2017 |
| ON986437 | Raccoon | USA:CT - Fairfield | 7/13/2017 |
| ON986438 | Raccoon | USA:CT - Fairfield | 9/21/2017 |
| ON986439 | Raccoon | USA:CT - Hartford | 9/27/2017 |
| ON986440 | Raccoon | USA:CT - Fairfield | 9/29/2017 |
| ON986441 | Raccoon | USA:CT - NewHaven | 12/1/2017 |
| ON986442 | Raccoon | USA:CT - Tolland | 2/10/2018 |
| ON986443 | Raccoon | USA:CT - Fairfield | 2/21/2018 |
| ON986444 | Raccoon | USA:CT - Tolland | 2/21/2018 |
| ON986445 | Raccoon | USA:CT - NewHaven | 2/24/2018 |
| ON986446 | Raccoon | USA:CT - Litchfield | 2/27/2018 |
| ON986447 | Raccoon | USA:CT - NewLondon | 3/19/2018 |
| ON986448 | Raccoon | USA:CT - NewLondon | 3/24/2018 |
| ON986449 | Raccoon | USA:CT - Tolland | 3/26/2018 |
| ON986450 | Raccoon | USA:CT - Fairfield | 6/19/2018 |
| ON986451 | Raccoon | USA:CT - Litchfield | 6/21/2018 |
| ON986452 | Raccoon | USA:CT - Fairfield | 7/5/2018 |
| ON986453 | Raccoon | USA:CT - NewHaven | 7/27/2018 |
| ON986454 | Raccoon | USA:CT - Fairfield | 9/10/2018 |
| ON986455 | Raccoon | USA:CT - Tolland | 9/12/2018 |
| ON986456 | Raccoon | USA:CT - Litchfield | 11/16/2018 |
| ON986457 | Raccoon | USA:CT - Tolland | 12/4/2018 |
| ON986458 | Raccoon | USA:CT - Middlesex | 2/2/2019 |
| ON986459 | Raccoon | USA:CT - Fairfield | 3/22/2019 |
| ON986460 | Raccoon | USA:CT - Fairfield | 4/3/2019 |
| ON986461 | Raccoon | USA:CT - Fairfield | 4/17/2019 |
| ON986462 | Raccoon | USA:CT - Windham | 5/9/2019 |
| ON986463 | Raccoon | USA:CT - NewLondon | 5/13/2019 |
| ON986464 | Raccoon | USA:CT - Tolland | 5/22/2019 |
| ON986465 | Raccoon | USA:CT - NewHaven | 6/12/2019 |
| ON986466 | Raccoon | USA:CT - Middlesex | 7/2/2019 |
| ON986467 | Raccoon | USA:CT - NewHaven | 8/22/2019 |
| ON986468 | Raccoon | USA:CT - Windham | 9/1/2019 |
| ON986469 | Raccoon | USA:CT - Windham | 9/25/2019 |
| ON986470 | Raccoon | USA:CT - Hartford | 10/24/2019 |
| ON986471 | Raccoon | USA:CT - Litchfield | 12/12/2019 |
| ON986472 | Raccoon | USA:CT - NewHaven | 12/12/2019 |
| ON986473 | Raccoon | USA:CT - Fairfield | 3/11/2020 |
| ON986474 | Skunk | USA:CT - Hartford | 2/6/2017 |
| ON986475 | Skunk | USA:CT - Windham | 7/10/2017 |
| ON986476 | Skunk | USA:CT - NewLondon | 7/31/2017 |
| ON986477 | Skunk | USA:CT - Windham | 9/5/2017 |
| ON986478 | Skunk | USA:CT - Middlesex | 5/30/2019 |
| ON986479 | Skunk | USA:CT - NewLondon | 8/23/2019 |
| ON986480 | Skunk | USA:CT - NewHaven | 11/1/2019 |
| ON986481 | Skunk | USA:CT - NewHaven | 12/3/2019 |
| ON986482 | Woodchuck | USA:CT - Windham | 8/30/2018 |
| ON986483 | Woodchuck | USA:CT - Middlesex | 11/1/2018 |
| ON986484 | Woodchuck | USA:CT - Windham | 6/17/2019 |
| MN418148 | Skunk | USA:CT - NewLondon | 10/1/2017 |
| MN418150 | Raccoon | USA:CT - NewLondon | 2/1/2016 |
| MN418151 | Raccoon | USA:CT - Fairfield | 7/6/2016 |
| MN418155 | Raccoon | USA:CT - NewHaven | 6/30/2016 |
| MN418160 | Raccoon | USA:CT - Fairfield | 5/1/2017 |
| MN418163 | Raccoon | USA:CT - Fairfield | 11/15/2016 |
| MN418181 | Raccoon | USA:CT - Fairfield | 12/14/2016 |
| MN418182 | Raccoon | USA:CT - Fairfield | 2/1/2016 |
| MK540729 | Procyonlotor | USA:NY | 2010 |
| MG562524 | Procyonlotor | USA:NY | 1990 |
| MK540707 | Procyonlotor | USA:NY | 2004 |
| MG562534 | Procyonlotor | USA:NY | 2004 |
| MK540747 | Foxes | USA:NY | 2011 |
| MG562528 | Procyonlotor | USA:NY | 2003 |
| MK540720 | Mephitidae | USA:NY | 2004 |
| MK540794 | Procyonlotor | USA:NY | 2015 |
| MK540796 | Procyonlotor | USA:NY | 2015 |
| MK540752 | Procyonlotor | USA:NY | 2011 |
| MN418145 | Procyonlotor | USA:NY | 3/3/2017 |
| MK540742 | Mephitidae | USA:NY | 2011 |
| MG562544 | Procyonlotor | USA:NY | 2010 |
| MK540786 | Foxes | USA:NY | 2011 |
| MG562530 | Procyonlotor | USA:NY | 2003 |
| MK540669 | Ruminants | USA:NY | 1990 |
| MG562538 | Procyonlotor | USA:NY | 2004 |
| MK540782 | Mephitidae | USA:NY | 2011 |
| MK540702 | Procyonlotor | USA:NY | 2004 |
| MN418144 | Procyonlotor | USA:NY | 12/3/2017 |
| MK540760 | Feliformia | USA:NY | 2011 |
| MK540769 | Feliformia | USA:NY | 2011 |
| MK540738 | Mephitidae | USA:NY | 2010 |
| MK540722 | Procyonlotor | USA:NY | 2009 |
| MK540671 | Procyonlotor | USA:NY | 1990 |
| MK540791 | Feliformia | USA:NY | 2011 |
| MK540711 | Procyonlotor | USA:NY | 2004 |
| MN418164 | Procyonlotor | USA:NY | 1/8/2009 |
| MK540759 | Feliformia | USA:NY | 2011 |
| MK540699 | Procyonlotor | USA:NY | 2004 |
| MG562525 | Procyonlotor | USA:NY | 1992 |
| MG562543 | Procyonlotor | USA:NY | 2010 |
| MK540772 | Feliformia | USA:NY | 2011 |
| MG562546 | Procyonlotor | USA:NY | 2010 |
| MK540746 | Procyonlotor | USA:NY | 2011 |
| MK540735 | Procyonlotor | USA:NY | 2010 |
| MK540713 | Mephitidae | USA:NY | 2004 |
| MK540692 | Procyonlotor | USA:NY | 2003 |
| MK540795 | Procyonlotor | USA:NY | 2015 |
| MG562537 | Procyonlotor | USA:NY | 2004 |
| MK540789 | Foxes | USA:NY | 2011 |
| MK540715 | Procyonlotor | USA:NY | 2004 |
| MK540733 | Procyonlotor | USA:NY | 2010 |
| MK540693 | Procyonlotor | USA:NY | 2003 |
| MK540750 | Procyonlotor | USA:NY | 2011 |
| MN418158 | Foxes | USA:NY | 6/6/2017 |
| MK540781 | Rodents | USA:NY | 2011 |
| MN418147 | Procyonlotor | USA:NY | 8/11/2017 |
| MK540682 | Procyonlotor | USA:NY | 1992 |
| MK540753 | Procyonlotor | USA:NY | 2011 |
| PP447323 | Procyonlotor | USA:VT | 3/14/2022 |
| PP447324 | Mephitidae | USA:VT | 12/21/2021 |
| PP447326 | Procyonlotor | USA:ME | 9/20/2021 |
| PP447327 | Procyonlotor | USA:ME | 9/2/2021 |
| PP447331 | Procyonlotor | USA:ME | 9/7/2021 |
| MN418146 | Procyonlotor | USA:VT | 8/29/2017 |
| MN418165 | Foxes | USA:VT | 3/3/2017 |
| MN418175 | Procyonlotor | USA:VT | 4/21/2017 |
| MN418177 | Foxes | USA:VT | 6/20/2017 |
| MN418179 | Procyonlotor | USA:VT | 8/19/2017 |
| MG562581 | Procyonlotor | USA:VT | 2008 |
| MG562582 | Mephitidae | USA:VT | 2008 |
| MG562602 | Procyonlotor | USA:VT | 2009 |
| MG562606 | Procyonlotor | USA:VT | 2009 |
| MG562607 | Foxes | USA:VT | 2010 |
| MG562610 | Mephitidae | USA:VT | 2011 |
| MF143191 | Mephitidae | USA:ME | 1/3/2013 |
| MF143192 | Procyonlotor | USA:ME | 2/4/2013 |
| MF143193 | Procyonlotor | USA:ME | 2/13/2013 |
| MF143195 | Procyonlotor | USA:ME | 3/21/2013 |
| MF143196 | Procyonlotor | USA:ME | 3/22/2013 |
| MF143197 | Mephitidae | USA:ME | 4/12/2013 |
| MF143198 | Mephitidae | USA:ME | 4/17/2013 |
| MF143200 | Procyonlotor | USA:ME | 4/25/2013 |
| MF143201 | Procyonlotor | USA:ME | 4/26/2013 |
| MF143202 | Mephitidae | USA:ME | 4/30/2013 |
| MF143203 | Foxes | USA:ME | 6/6/2013 |
| MF143205 | Procyonlotor | USA:ME | 6/25/2013 |
| MF143206 | Procyonlotor | USA:ME | 7/19/2013 |
| MF143207 | Foxes | USA:ME | 7/31/2013 |
| MF143208 | Procyonlotor | USA:ME | 8/19/2013 |
| MF143209 | Mephitidae | USA:ME | 10/11/2013 |
| MF143210 | Mephitidae | USA:ME | 10/28/2013 |
| MF143211 | Mephitidae | USA:ME | 12/20/2013 |
| MF143212 | Mephitidae | USA:ME | 12/31/2013 |
| MF143213 | Procyonlotor | USA:ME | 2/27/2014 |
| MF143215 | Procyonlotor | USA:ME | 5/2/2014 |
| MF143216 | Mephitidae | USA:ME | 5/7/2014 |
| MF143218 | Procyonlotor | USA:ME | 5/13/2014 |
| MF143219 | Procyonlotor | USA:ME | 5/22/2014 |
| MF143220 | Foxes | USA:ME | 6/13/2014 |
| MF143278 | Ruminants | USA:VT | 9/5/2006 |
| MF143279 | Procyonlotor | USA:VT | 10/11/2006 |
| MF143280 | Mephitidae | USA:VT | 10/30/2006 |
| MF143281 | Mephitidae | USA:VT | 5/23/2006 |
| MF143282 | Procyonlotor | USA:VT | 7/25/2007 |
| MF143283 | Procyonlotor | USA:VT | 1/4/2007 |
| MF143284 | Procyonlotor | USA:VT | 1/31/2007 |
| MF143285 | Mephitidae | USA:VT | 9/11/2007 |
| MF143286 | Procyonlotor | USA:VT | 3/30/2007 |
| MF143287 | Procyonlotor | USA:VT | 4/25/2007 |
| MF143288 | Mephitidae | USA:VT | 11/6/2008 |
| MF143289 | Procyonlotor | USA:VT | 3/17/2009 |
| MF143337 | Mephitidae | USA:VT | 11/5/2007 |
| MF143338 | Ruminants | USA:VT | 4/5/2009 |
| MF143339 | Mephitidae | USA:VT | 11/18/2010 |
| MF143340 | Mephitidae | USA:VT | 11/4/2011 |
| MF143373 | Procyonlotor | USA:VT | 12/29/2005 |
| MF143374 | Procyonlotor | USA:VT | 8/29/2006 |
| MF143375 | Procyonlotor | USA:VT | 8/30/2006 |
| MF143376 | Mephitidae | USA:VT | 9/5/2006 |
| MF143377 | Mephitidae | USA:VT | 10/4/2006 |
| MF143378 | Mephitidae | USA:VT | 7/20/2007 |
| MF143379 | Procyonlotor | USA:VT | 3/2/2007 |
| MF143380 | Procyonlotor | USA:VT | 9/18/2007 |
| MF143381 | Procyonlotor | USA:VT | 3/22/2007 |
| MF143382 | Mephitidae | USA:VT | 9/26/2007 |
| MF143383 | Mephitidae | USA:VT | 10/15/2007 |
| MF143384 | Mephitidae | USA:VT | 10/30/2007 |
| MF143385 | Procyonlotor | USA:VT | 11/5/2007 |
| MF143386 | Procyonlotor | USA:VT | 4/25/2007 |
| MF143387 | Mephitidae | USA:VT | 4/30/2007 |
| MF143388 | Mephitidae | USA:VT | 11/14/2007 |
| MF143389 | Mephitidae | USA:VT | 11/20/2007 |
| MF143390 | Mephitidae | USA:VT | 12/10/2007 |
| MF143391 | Ruminants | USA:VT | 12/29/2008 |
| MF143392 | Mephitidae | USA:VT | 7/20/2009 |
| MF143393 | Procyonlotor | USA:VT | 8/14/2009 |
| MF143394 | Mephitidae | USA:VT | 8/20/2009 |
| MF143395 | Mephitidae | USA:VT | 10/4/2009 |
| MF143396 | Mephitidae | USA:VT | 10/12/2009 |
| MF143397 | Procyonlotor | USA:VT | 10/19/2009 |
| MF143398 | Procyonlotor | USA:VT | 10/19/2009 |
| MF143399 | Procyonlotor | USA:VT | 11/5/2009 |
| MF143400 | Mephitidae | USA:VT | 11/1/2009 |
| MF143401 | Procyonlotor | USA:VT | 11/30/2009 |
| MF143402 | Procyonlotor | USA:VT | 2/26/2009 |
| MF143403 | Ruminants | USA:VT | 3/11/2009 |
| MF143404 | Procyonlotor | USA:VT | 4/17/2009 |
| MF143405 | Procyonlotor | USA:VT | 4/17/2009 |
| MF143406 | Ruminants | USA:VT | 5/12/2009 |
| MF143407 | Mephitidae | USA:VT | 7/29/2010 |
| MF143408 | Procyonlotor | USA:VT | 8/9/2010 |
| MF143409 | Procyonlotor | USA:VT | 8/20/2010 |
| MF143410 | Mephitidae | USA:VT | 8/23/2010 |
| MF143411 | Mephitidae | USA:VT | 8/29/2010 |
| MF143412 | Mephitidae | USA:VT | 10/14/2010 |
| MF143413 | Mephitidae | USA:VT | 6/18/2010 |
| MF143414 | Mephitidae | USA:VT | 8/27/2011 |
| MF143415 | Procyonlotor | USA:VT | 1/8/2011 |
| MF143273 | Procyonlotor | Canada:QC | 5/7/2008 |
| MF143323 | Mephitidae | USA:NY | 10/26/2010 |
| MK540861 | Mephitidae | Canada:ON | 2016 |
| MF143236 | Procyonlotor | Canada:NB | 1/22/2015 |
| MF143276 | Mephitidae | Canada:QC | 2/26/2009 |
| MF143267 | Procyonlotor | Canada:QC | 7/14/2007 |
| MF143269 | Procyonlotor | Canada:QC | 7/19/2007 |
| MK540809 | Procyonlotor | Canada:ON | 2016 |
| MK540678 | Procyonlotor | USA:NY | 1991 |
| MK540844 | Procyonlotor | Canada:ON | 2016 |
| MF143259 | Procyonlotor | Canada:QC | 8/1/2007 |
| KY026417 | Procyonlotor | USA:NY | 11/9/1998 |
| MK540815 | Procyonlotor | Canada:ON | 2016 |
| MF143221 | Procyonlotor | USA:ME | 6/26/2014 |
| MK540862 | Mephitidae | Canada:ON | 2016 |
| MK540863 | Mephitidae | Canada:ON | 2016 |
| MK540860 | Procyonlotor | Canada:ON | 2016 |
| MK540877 | Mephitidae | Canada:ON | 2017 |
| MF143227 | Procyonlotor | Canada:NB | 11/16/2000 |
| MK540850 | Procyonlotor | Canada:ON | 2016 |
| MF143265 | Procyonlotor | Canada:QC | 6/21/2007 |
| MK540808 | Procyonlotor | Canada:ON | 2016 |
| MK540856 | Procyonlotor | Canada:ON | 2016 |
| MF143352 | Procyonlotor | Canada:QC | 7/3/2007 |
| MK540709 | Procyonlotor | USA:NY | 2004 |
| MF143346 | Procyonlotor | Canada:QC | 8/30/2007 |
| MF143368 | Procyonlotor | Canada:QC | 8/25/2008 |
| MF143233 | Procyonlotor | Canada:NB | 8/22/2001 |
| MK540843 | Vulpesvulpes | Canada:ON | 2016 |
| MF143366 | Procyonlotor | Canada:QC | 6/16/2008 |
| MF143330 | Mephitidae | USA:NY | 9/8/2011 |
| MF143243 | Procyonlotor | Canada:NB | 3/18/2015 |
| MK540745 | Procyonlotor | USA:NY | 2011 |
| MK540827 | Procyonlotor | Canada:ON | 2016 |
| MK540821 | Mephitidae | Canada:ON | 2016 |
| MK540764 | Vulpesvulpes | USA:NY | 2011 |
| MF143251 | Mephitidae | Canada:NB | 7/29/2015 |
| MG562541 | Mephitidae | USA:NY | 2004 |
| MF143329 | Procyonlotor | USA:NY | 9/2/2011 |
| MF143245 | Procyonlotor | Canada:NB | 4/14/2015 |
| MF143299 | Procyonlotor | USA:NY | 5/4/2004 |
| MK540840 | Procyonlotor | Canada:ON | 2016 |
| MF143364 | Procyonlotor | Canada:QC | 5/13/2008 |
| MK540858 | Mephitidae | Canada:ON | 2016 |
| MF143204 | Mephitidae | USA:ME | 6/10/2013 |
| MF143266 | Procyonlotor | Canada:QC | 6/29/2007 |
| MF143247 | Procyonlotor | Canada:NB | 5/7/2015 |
| MK540871 | Mephitidae | Canada:ON | 2016 |
| MK540806 | Procyonlotor | Canada:ON | 2016 |
| MK540807 | Procyonlotor | Canada:ON | 2016 |
| MF143359 | Vulpesvulpes | Canada:QC | 11/15/2007 |
| MK540876 | Procyonlotor | Canada:ON | 2017 |
| MF143264 | Procyonlotor | Canada:QC | 6/16/2007 |
| MK540696 | Mephitidae | USA:NY | 2004 |
| MF143229 | Mephitidae | Canada:NB | 1/2/2001 |
| MK540818 | Procyonlotor | Canada:ON | 2016 |
| MK540853 | Procyonlotor | Canada:ON | 2016 |
| MK540788 | Mephitidae | USA:NY | 2011 |
| MF143223 | Mephitidae | Canada:NB | 9/12/2000 |
| MF143344 | Procyonlotor | Canada:QC | 8/2/2007 |
| MF143369 | Procyonlotor | Canada:QC | 9/3/2008 |
| MK540866 | Mephitidae | Canada:ON | 2016 |
| MK540874 | Procyonlotor | Canada:ON | 2017 |
| MK540875 | Procyonlotor | Canada:ON | 2017 |
| MK540872 | Procyonlotor | Canada:ON | 2017 |
| MF143249 | Procyonlotor | Canada:NB | 7/14/2015 |
| MK540814 | Mephitidae | Canada:ON | 2016 |
| MK540703 | Procyonlotor | USA:NY | 2004 |
| MF143194 | Mephitidae | USA:ME | 2/28/2013 |
| MN418184 | Procyonlotor | USA:ME | 12/15/2017 |
| MF143217 | Procyonlotor | USA:ME | 5/8/2014 |
| MK540829 | Procyonlotor | Canada:ON | 2016 |
| MK540868 | Procyonlotor | Canada:ON | 2016 |
| MK540831 | Procyonlotor | Canada:ON | 2016 |
| MF143246 | Procyonlotor | Canada:NB | 5/5/2015 |
| MK540855 | Procyonlotor | Canada:ON | 2016 |
| MK540820 | Procyonlotor | Canada:ON | 2016 |
| MK540838 | Procyonlotor | Canada:ON | 2016 |
| MF143225 | Procyonlotor | Canada:NB | 11/7/2000 |
| MK540763 | Procyonlotor | USA:NY | 2011 |
| MF143277 | Procyonlotor | Canada:QC | 6/4/2015 |
| MK540778 | Procyonlotor | USA:NY | 2011 |
| MK540706 | Procyonlotor | USA:NY | 2004 |
| MK540836 | Mephitidae | Canada:ON | 2016 |
| MF143271 | Mephitidae | Canada:QC | 10/12/2007 |
| MK540837 | Procyonlotor | Canada:ON | 2016 |
| MF143224 | Mephitidae | Canada:NB | 10/31/2000 |
| MF143354 | Procyonlotor | Canada:QC | 8/23/2007 |
| KY026476 | Procyonlotor | Canada:ON | 9/22/2004 |
| MF143274 | Procyonlotor | Canada:QC | 5/8/2008 |
| MK540880 | Mephitidae | Canada:ON | 2017 |
| MK540803 | Procyonlotor | Canada:ON | 2015 |
| MF143239 | Procyonlotor | Canada:NB | 3/4/2015 |
| MK540846 | Procyonlotor | Canada:ON | 2016 |
| MF143214 | Mephitidae | USA:ME | 4/22/2014 |
| MK540802 | Procyonlotor | Canada:ON | 2015 |
| MF143353 | Procyonlotor | Canada:QC | 7/5/2007 |
| MK540787 | Mephitidae | USA:NY | 2011 |
| MK540824 | Mephitidae | Canada:ON | 2016 |
| MF143270 | Procyonlotor | Canada:QC | 8/3/2007 |
| MK540805 | Procyonlotor | Canada:ON | 2016 |
| MF143365 | Procyonlotor | Canada:QC | 5/13/2008 |
| MF143235 | Procyonlotor | Canada:NB | 6/2/2014 |
| MF143303 | Procyonlotor | USA:NY | 8/13/2004 |
| MF143262 | Procyonlotor | Canada:QC | 6/13/2007 |
| MF143341 | Procyonlotor | Canada:QC | 6/2/2006 |
| MK540845 | Mephitidae | Canada:ON | 2016 |
| KY026437 | Procyonlotor | Canada:ON | 6/19/2000 |
| MK540761 | Feliscatus | USA:NY | 2011 |
| MF143244 | Procyonlotor | Canada:NB | 3/19/2015 |
| MK540757 | Urocyoncinereoargenteus | USA:NY | 2011 |
| MK540830 | Procyonlotor | Canada:ON | 2016 |
| MK540826 | Procyonlotor | Canada:ON | 2016 |
| MK540819 | Mephitidae | Canada:ON | 2016 |
| MF143324 | Procyonlotor | USA:NY | 4/5/2011 |
| MK540701 | Procyonlotor | USA:NY | 2004 |
| MK540833 | Mephitidae | Canada:ON | 2016 |
| MF143242 | Procyonlotor | Canada:NB | 3/18/2015 |
| MF143350 | Procyonlotor | Canada:QC | 6/13/2007 |
| MF143294 | Mephitidae | USA:NY | 11/24/2003 |
| MF143371 | Procyonlotor | Canada:QC | 9/20/2008 |
| MK540817 | Mephitidae | Canada:ON | 2016 |
| MF143263 | Procyonlotor | Canada:QC | 6/14/2007 |
| MF143226 | Mephitidae | Canada:NB | 11/16/2000 |
| MF143311 | Mephitidae | USA:NY | 4/23/2010 |
| PP447322 | Procyonlotor | USA:ME | 1/4/2021 |
| MF143260 | Procyonlotor | Canada:QC | 5/11/2007 |
| MK540842 | Procyonlotor | Canada:ON | 2016 |
| MK540721 | Procyonlotor | USA:NY | 2004 |
| MK540804 | Procyonlotor | Canada:ON | 2015 |
| MK540834 | Procyonlotor | Canada:ON | 2016 |
| MF143199 | Procyonlotor | USA:ME | 4/17/2013 |
| MF143275 | Mephitidae | Canada:QC | 10/15/2008 |
| MK540762 | Feliscatus | USA:NY | 2011 |
| MF143248 | Procyonlotor | Canada:NB | 6/23/2015 |
| MK540857 | Procyonlotor | Canada:ON | 2016 |
| MK540881 | Mephitidae | Canada:ON | 2017 |
| MK540811 | Procyonlotor | Canada:ON | 2016 |
| MF143363 | Procyonlotor | Canada:QC | 4/15/2008 |
| MF143355 | Procyonlotor | Canada:QC | 9/21/2007 |
| MK540867 | Mephitidae | Canada:ON | 2016 |
| MF143347 | Procyonlotor | Canada:QC | 6/13/2007 |
| MF143351 | Procyonlotor | Canada:QC | 6/13/2007 |
| MF143234 | Procyonlotor | Canada:NB | 5/30/2002 |
| MK540816 | Mephitidae | Canada:ON | 2016 |
| MK540813 | Procyonlotor | Canada:ON | 2016 |
| MN418180 | Mephitismephitis | USA:ME | 12/6/2017 |
| MF143252 | Procyonlotor | Canada:NB | 8/6/2015 |
| MF143241 | Procyonlotor | Canada:NB | 3/10/2015 |
| MK540800 | Procyonlotor | Canada:ON | 2015 |
| MF143240 | Procyonlotor | Canada:NB | 3/10/2015 |
| MF143308 | Mephitidae | USA:NY | 4/8/2010 |
| MF143345 | Procyonlotor | Canada:QC | 8/29/2007 |
| MK540841 | Mephitidae | Canada:ON | 2016 |
| MF143268 | Procyonlotor | Canada:QC | 7/15/2007 |
| MK540870 | Mephitidae | Canada:ON | 2016 |
| MK540839 | Procyonlotor | Canada:ON | 2016 |
| MF143253 | Procyonlotor | Canada:NB | 8/18/2015 |
| MF143261 | Procyonlotor | Canada:QC | 6/11/2007 |
| MF143256 | Procyonlotor | Canada:QC | 10/20/2007 |

**S Table 11. Metadata of all sequences included in this study.**
