# Supplementary figures and images for: Molecular epidemiology, evolution, and transmission dynamics of raccoon rabies virus in Connecticut
Source: Virus Evol. 2024 Dec 24;11(1):veae114. doi: 10.1093/ve/veae114 (PMC11711587; doi:10.1093/ve/veae114)

**A**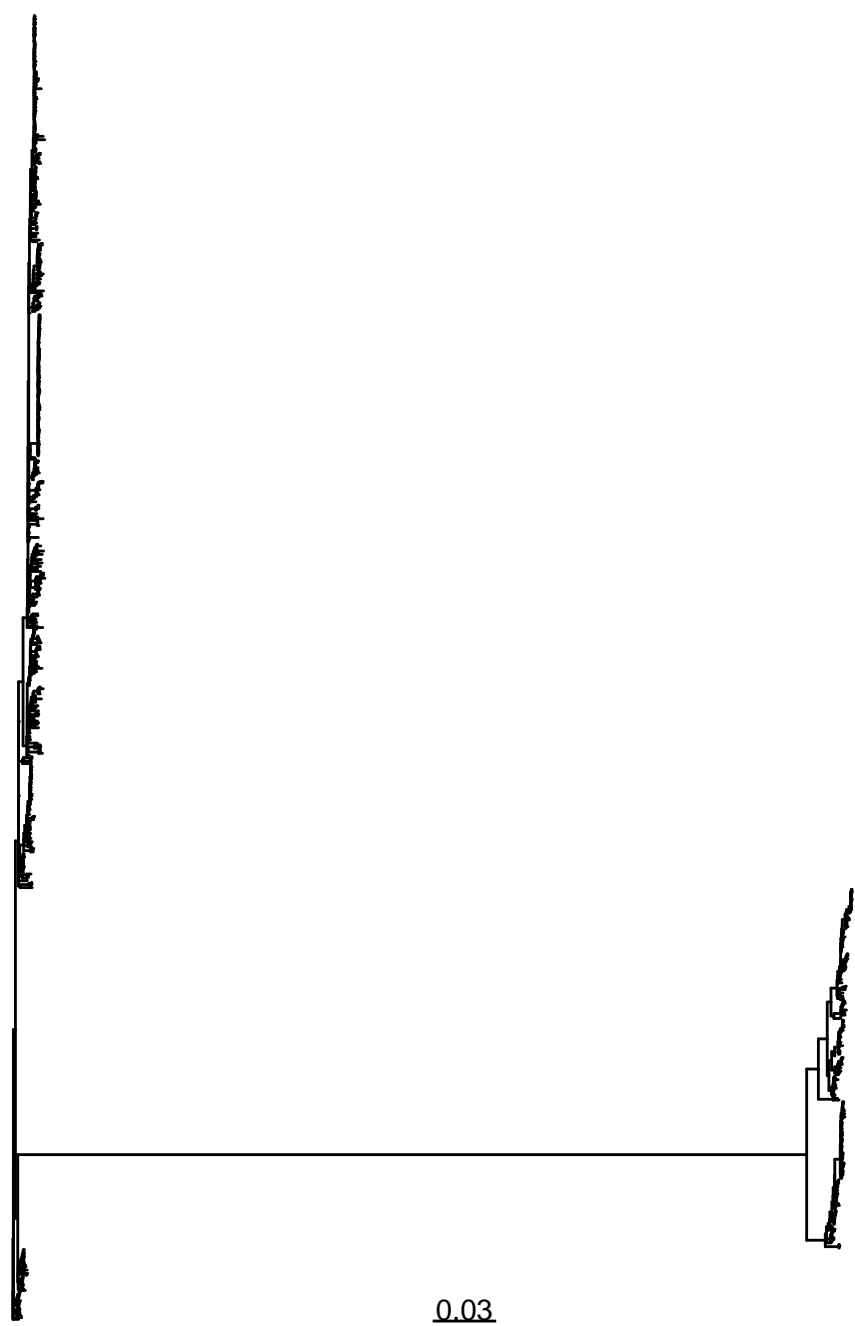**B**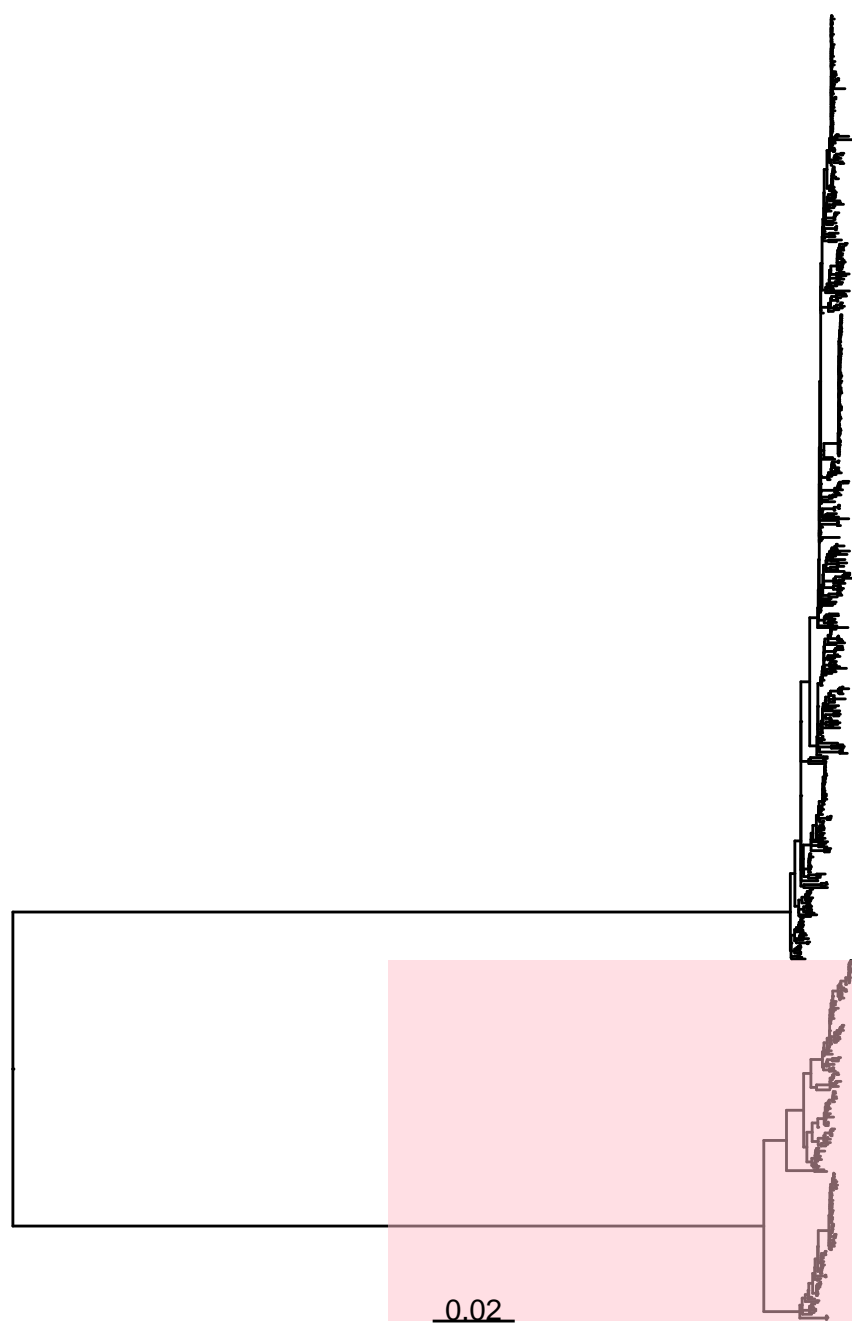

Supplement: veae114_Supp [file veae114_supp.zip › suppl_data/S Figure 1. Maximum likelihood phylogenetic tree of 768 North American sequences.pdf]

Clade 1

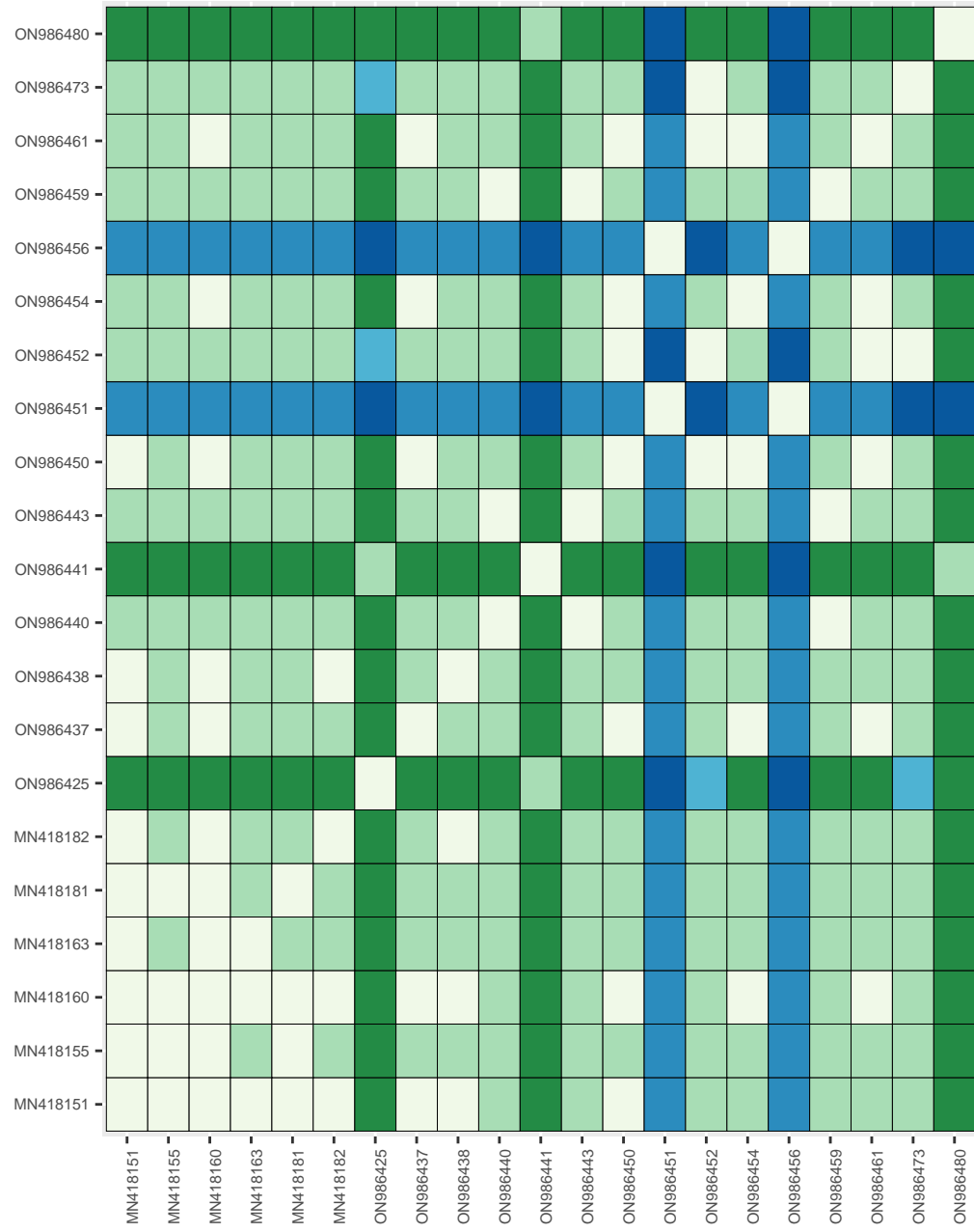

Clade 2

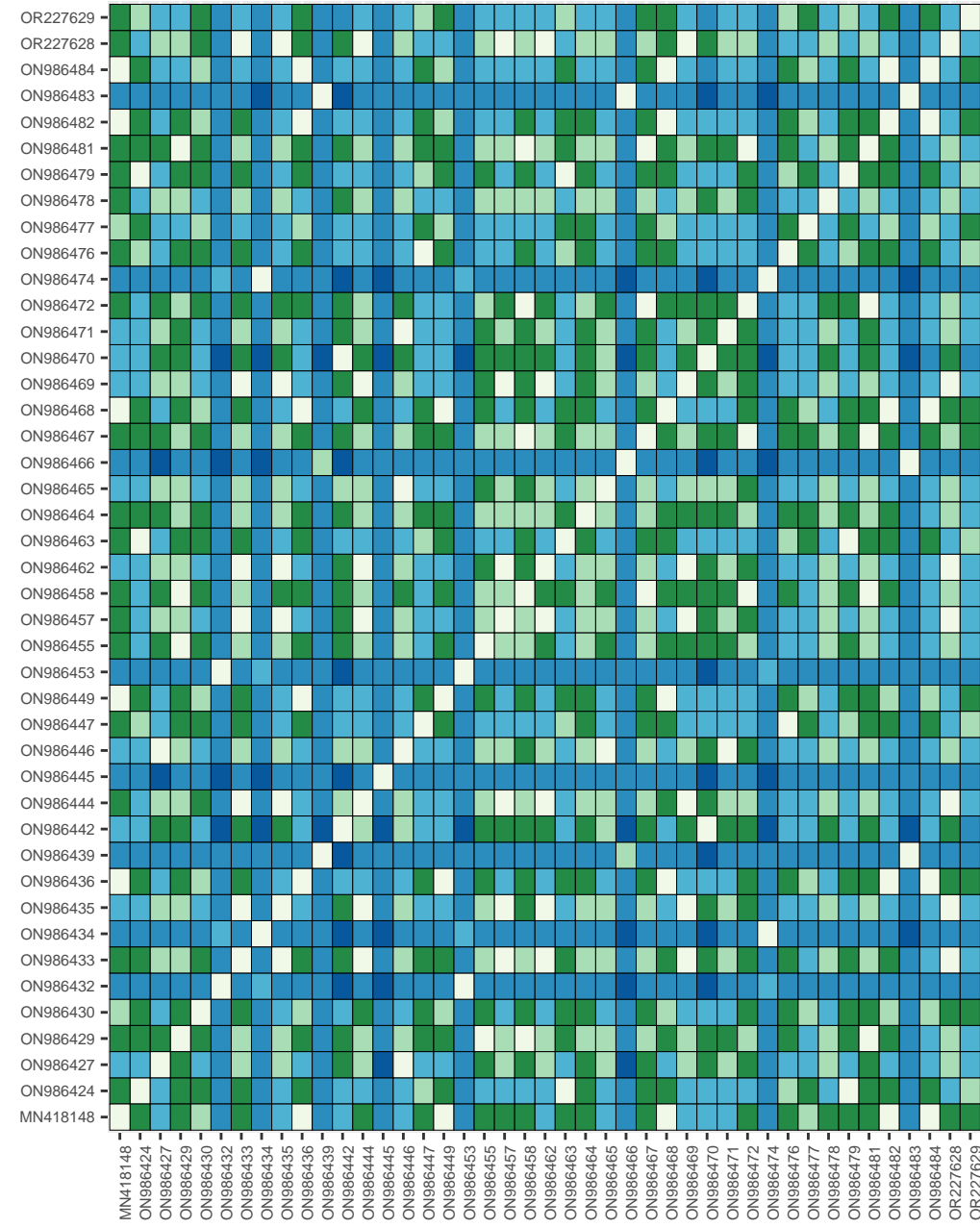

Clade 1/2

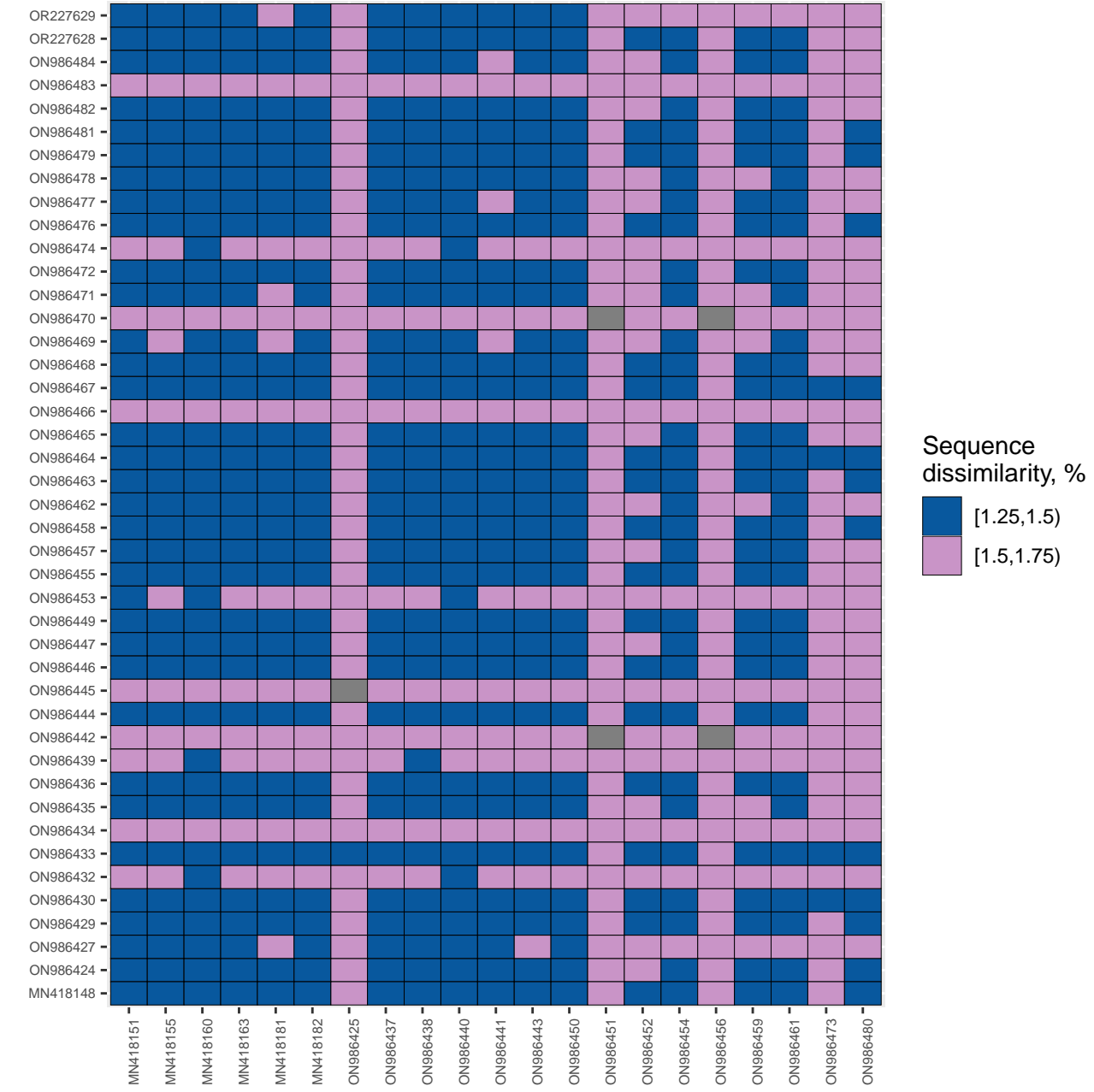

Supplement: veae114_Supp [file veae114_supp.zip › suppl_data/S Figure 10. RRV pairwise genetic distance between isolates, Clades.pdf]

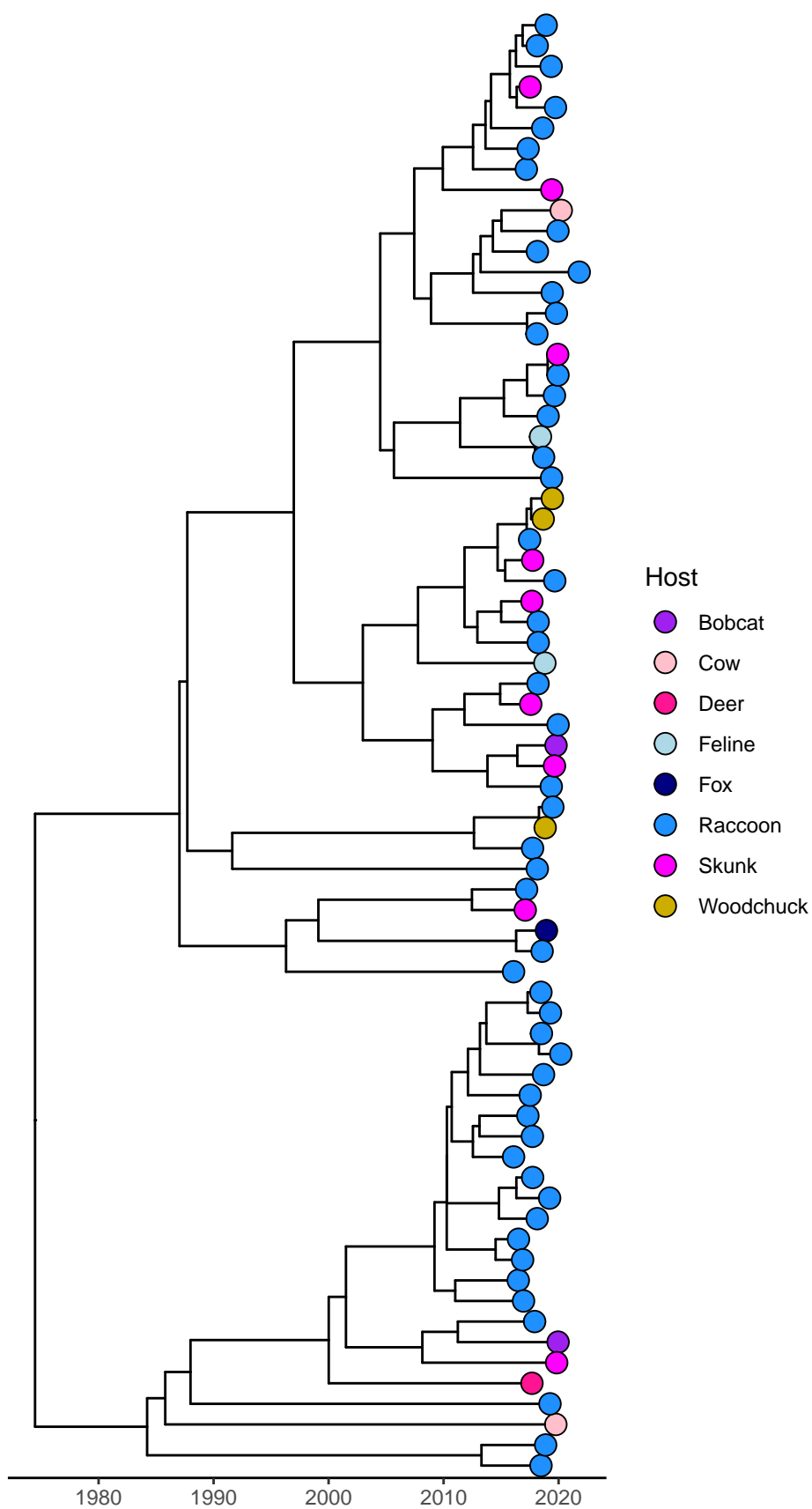

Supplement: veae114_Supp [file veae114_supp.zip › suppl_data/S Figure 11. Phylogenetic tree of 71 Connecticut sequences, Host.pdf]

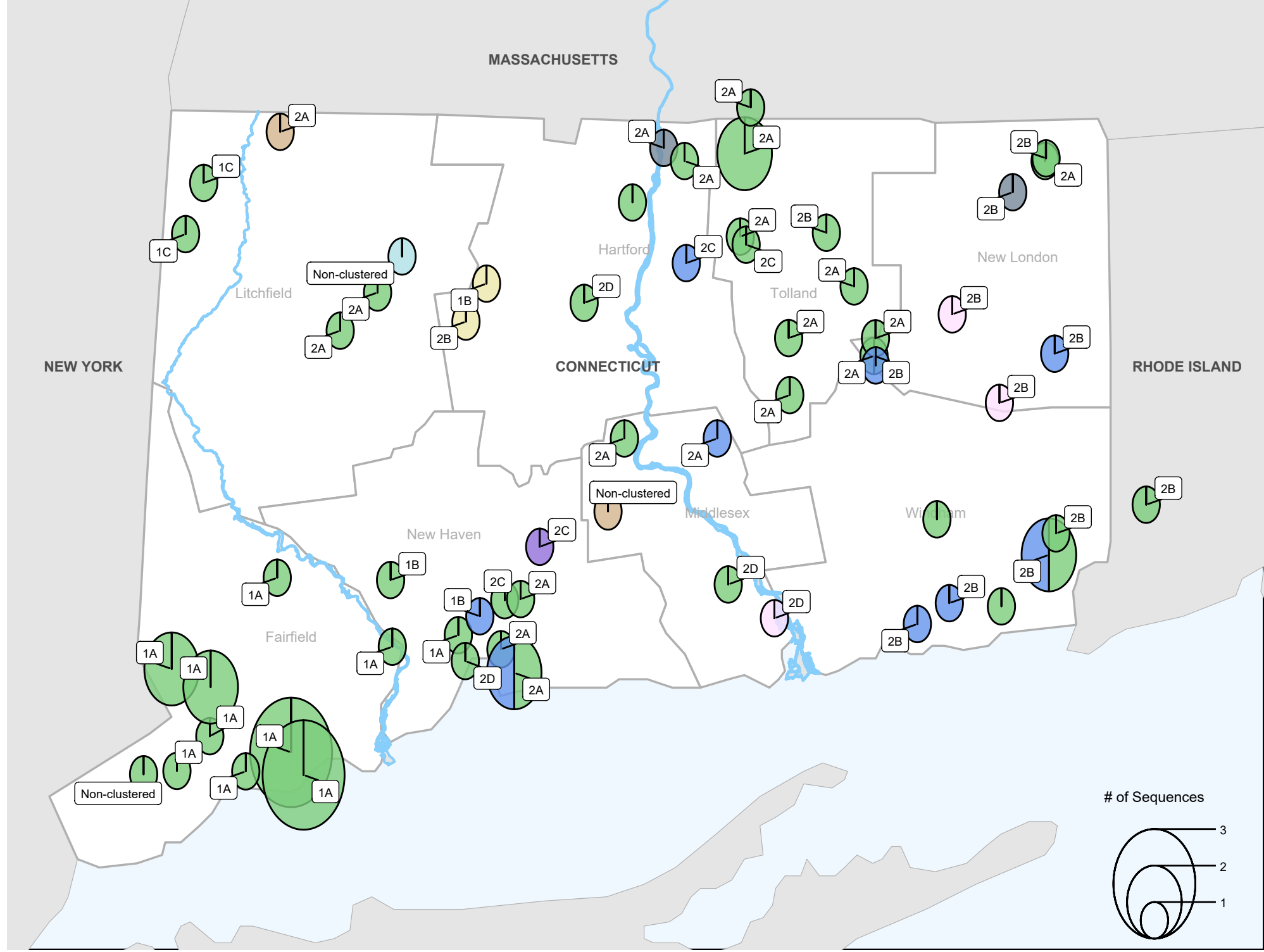

Supplement: veae114_Supp [file veae114_supp.zip › suppl_data/S Figure 12. Distribution of RRV variants in Connecticut, Species.pdf]

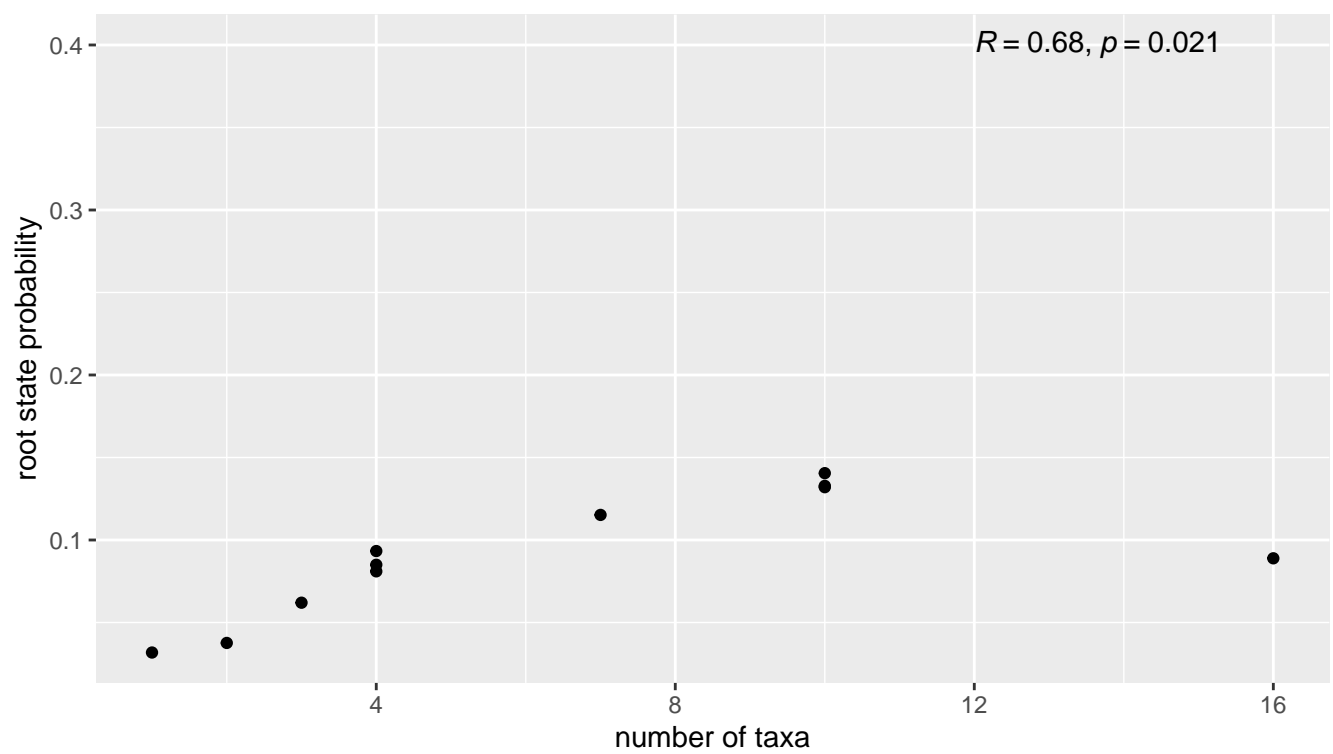

Supplement: veae114_Supp [file veae114_supp.zip › suppl_data/S Figure 13. Tip Trait Randomization, Connecticut.pdf]

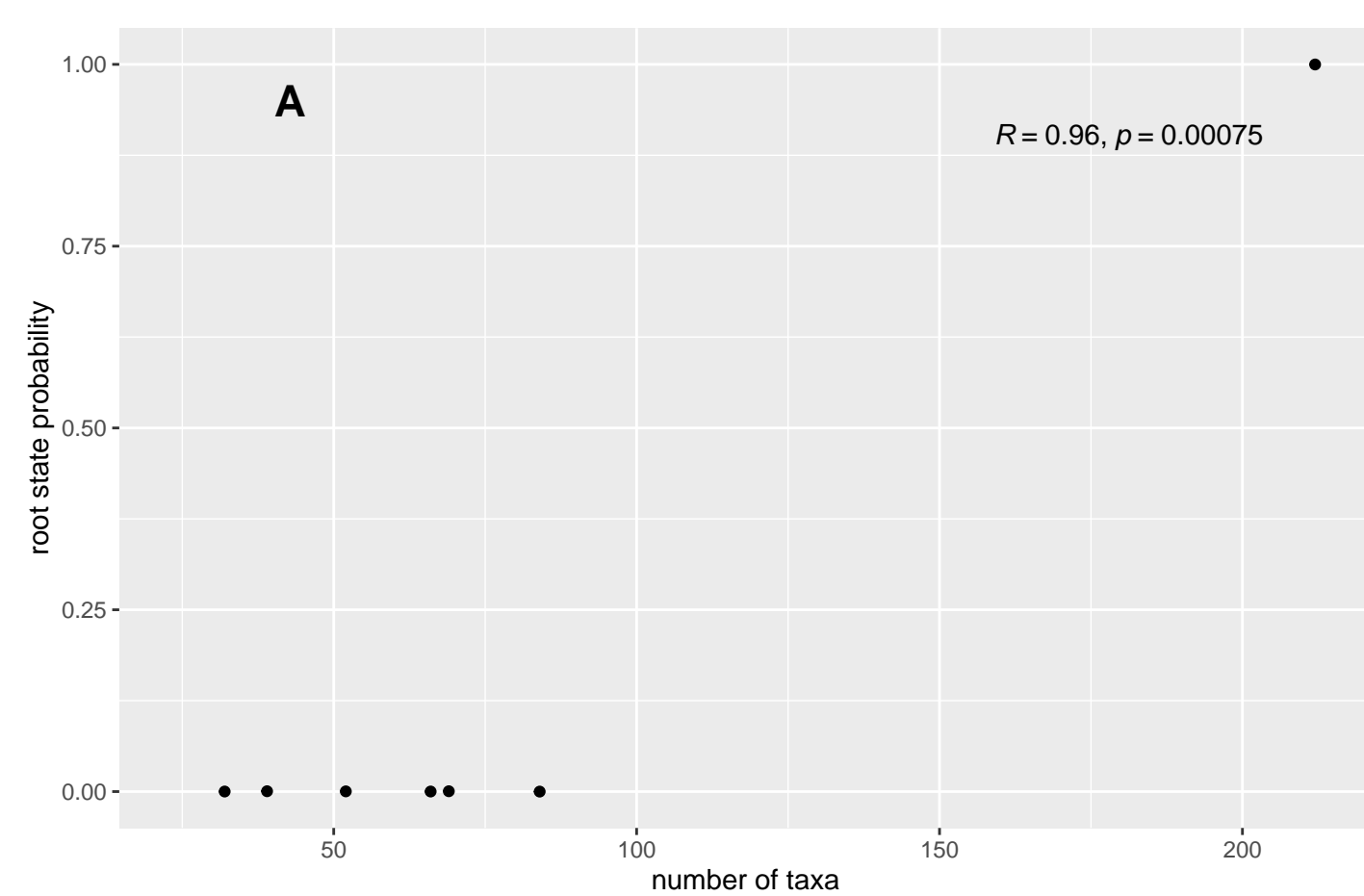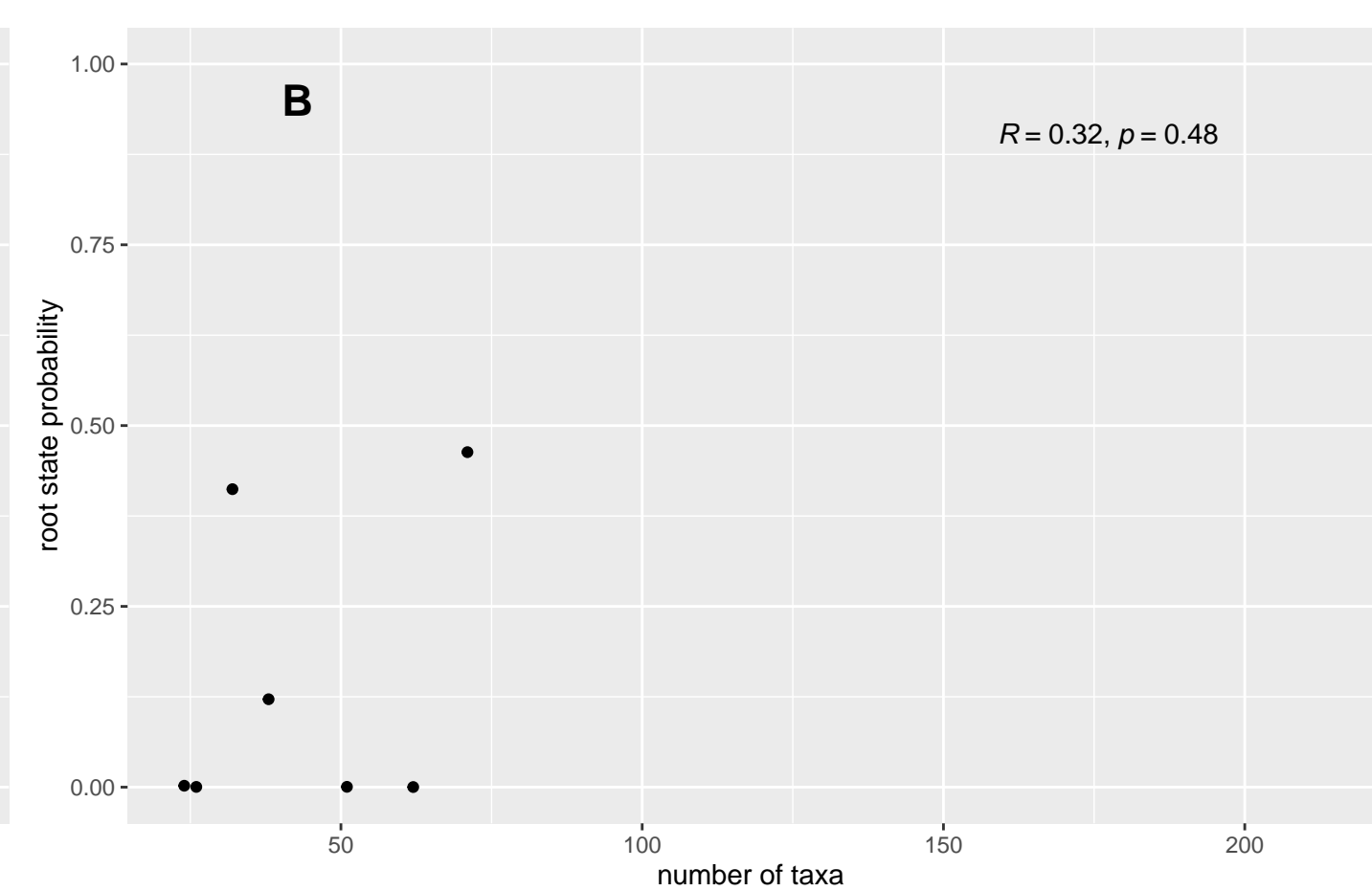

Supplement: veae114_Supp [file veae114_supp.zip › suppl_data/S Figure 2. Tip Trait Randomization, North America.pdf]

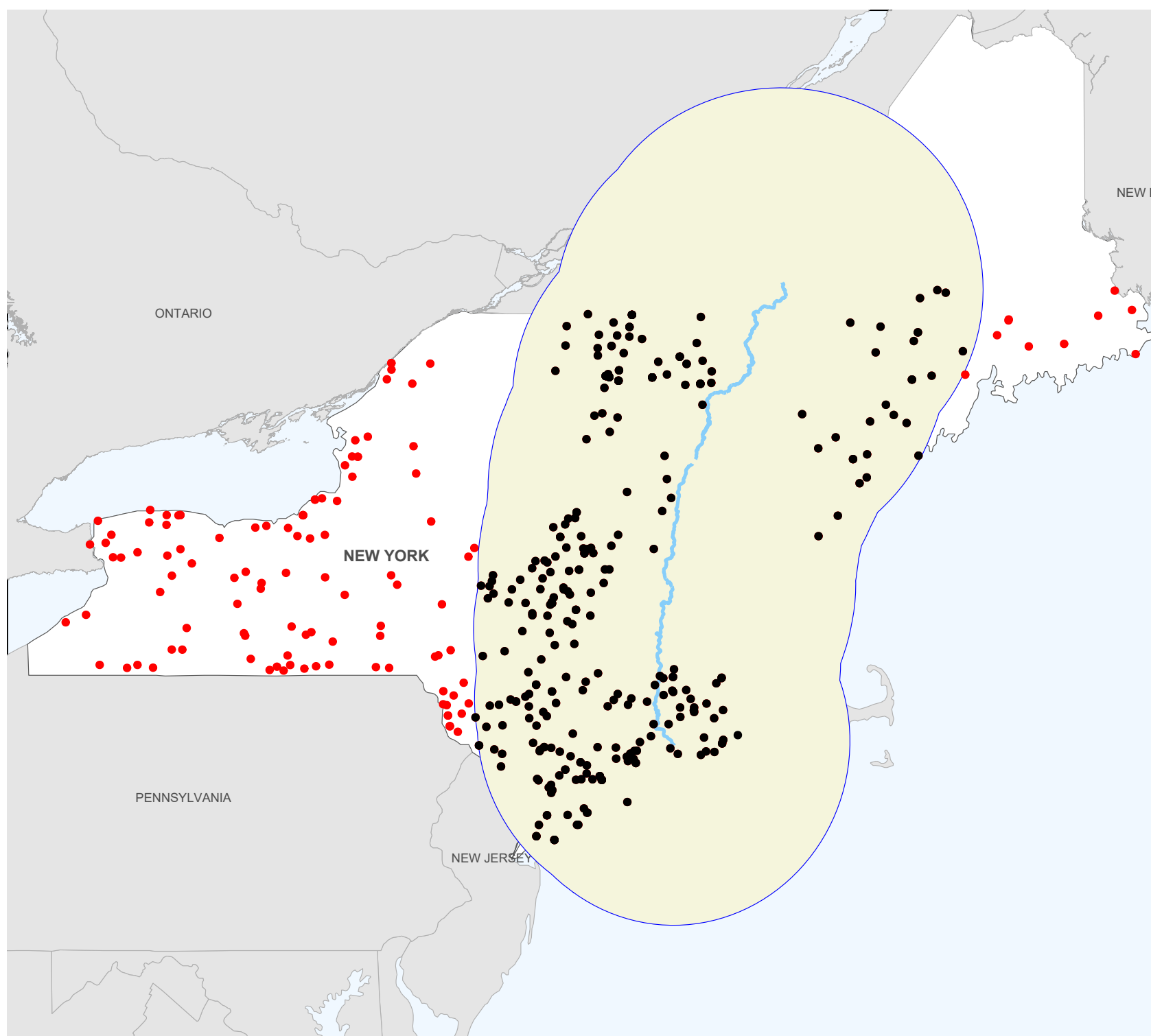

Supplement: veae114_Supp [file veae114_supp.zip › suppl_data/S Figure 3. Connecticut River Buffer.pdf]

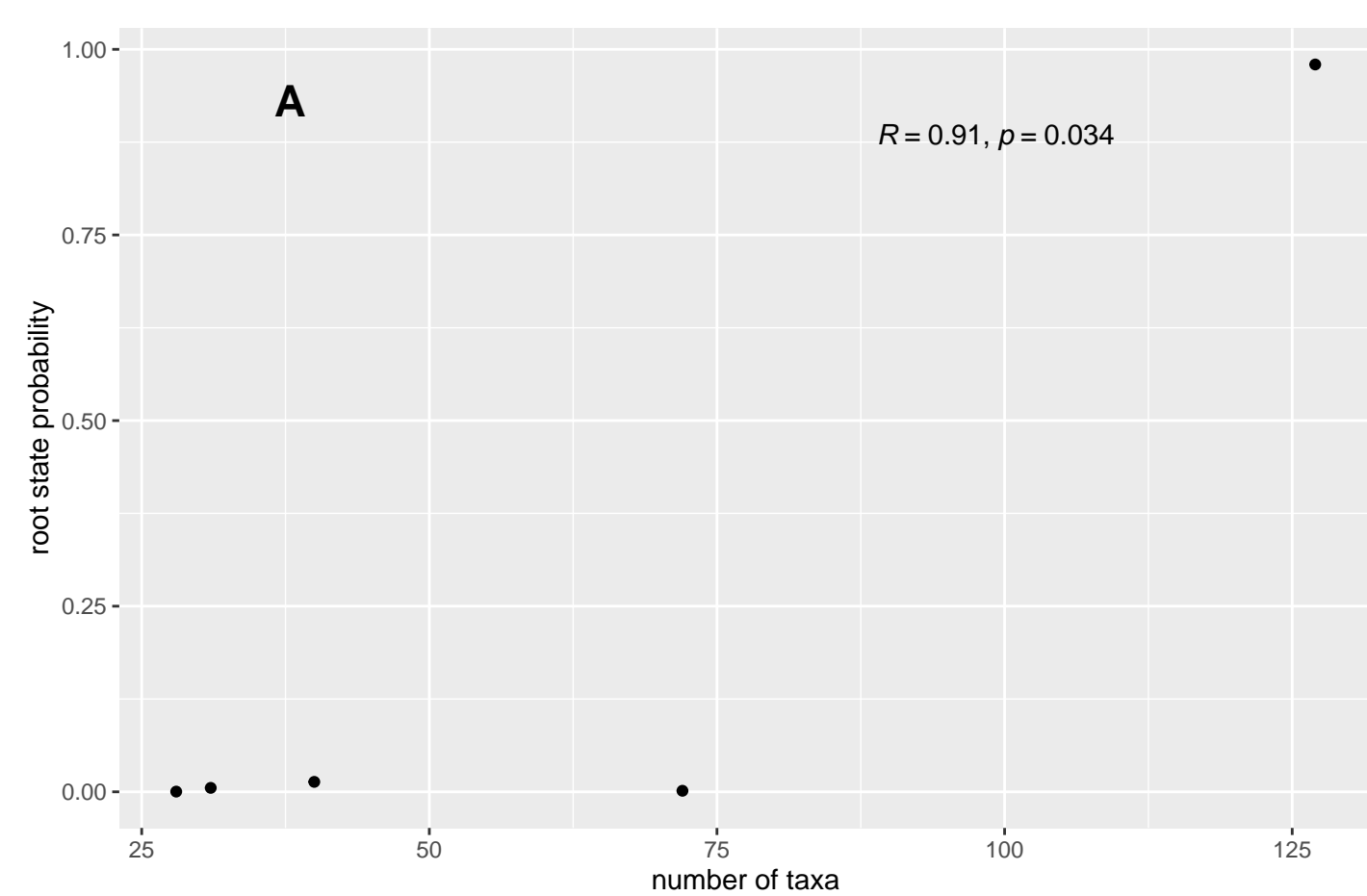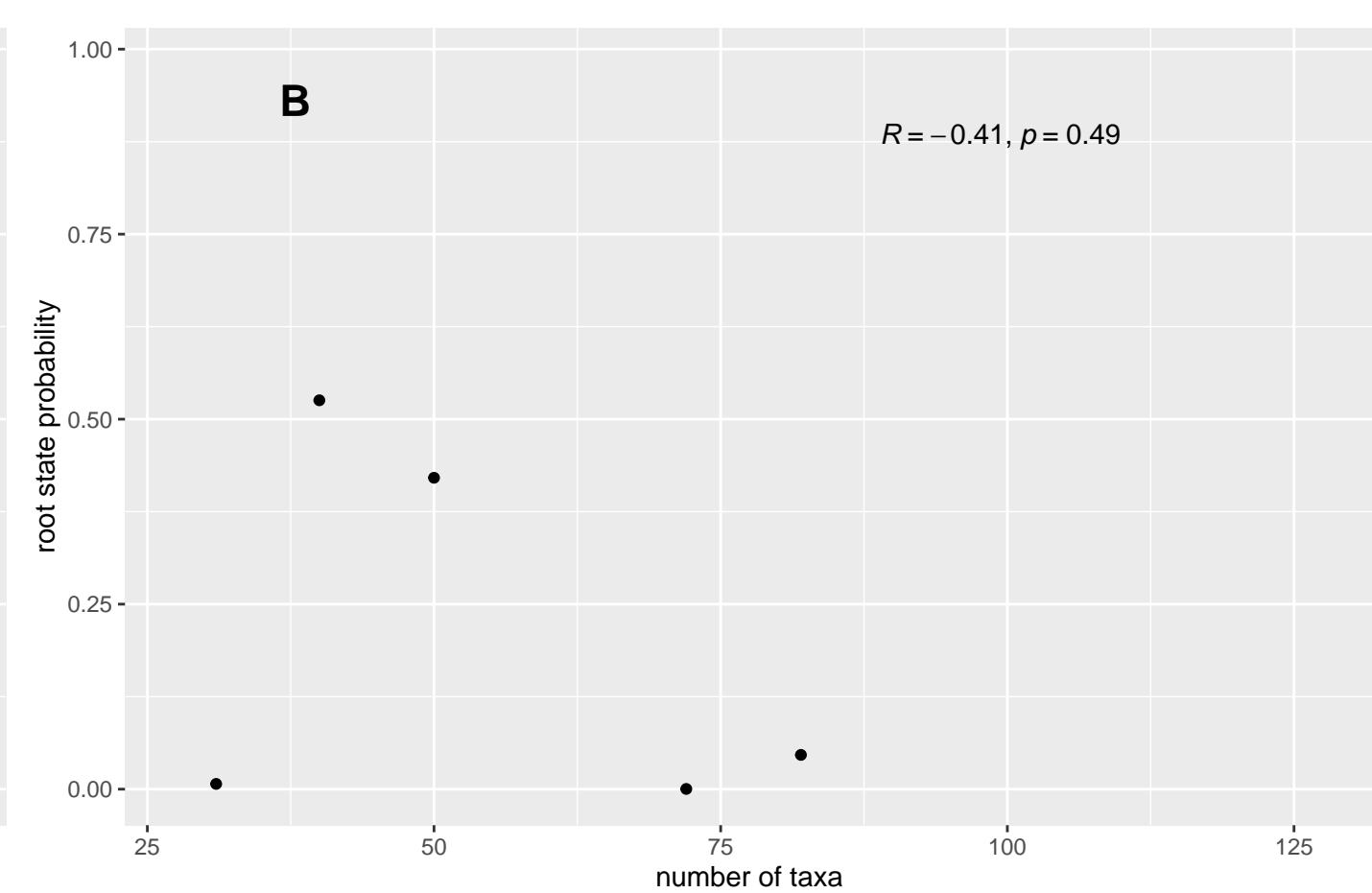

Supplement: veae114_Supp [file veae114_supp.zip › suppl_data/S Figure 4. Tip Trait Randomization, Connecticut River.pdf]

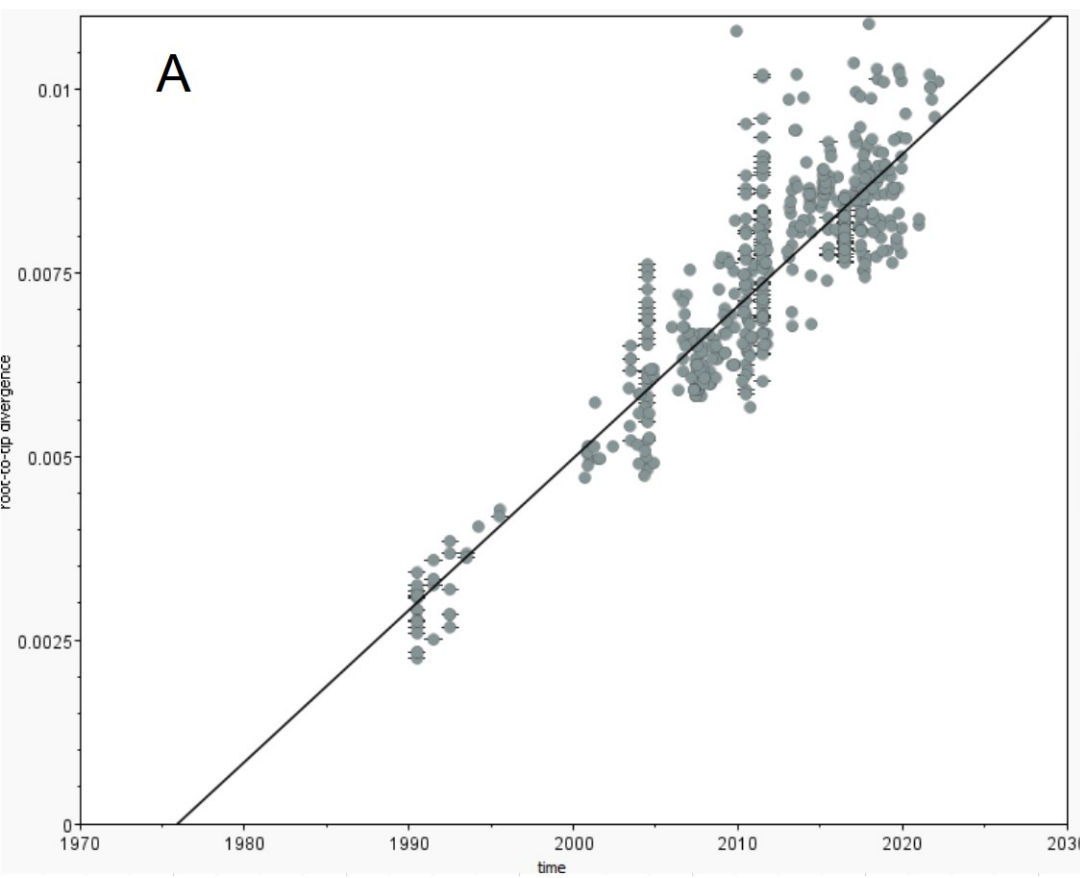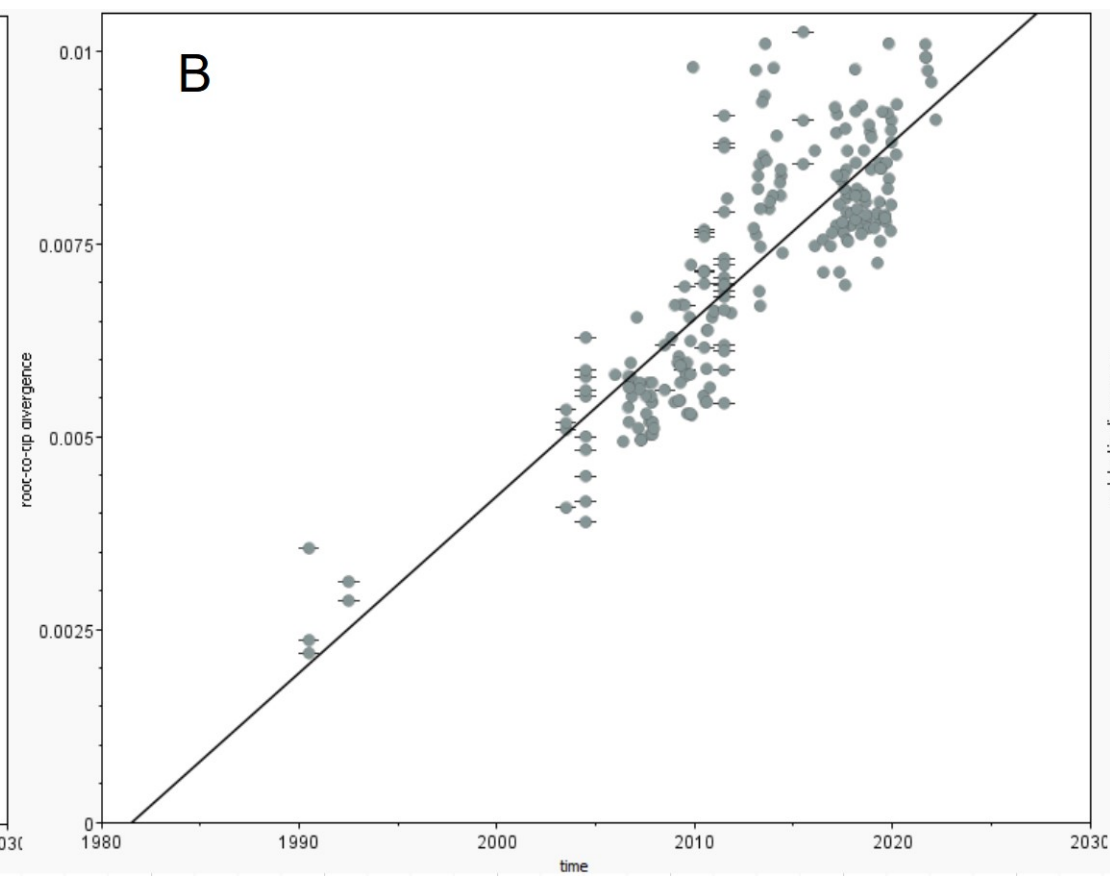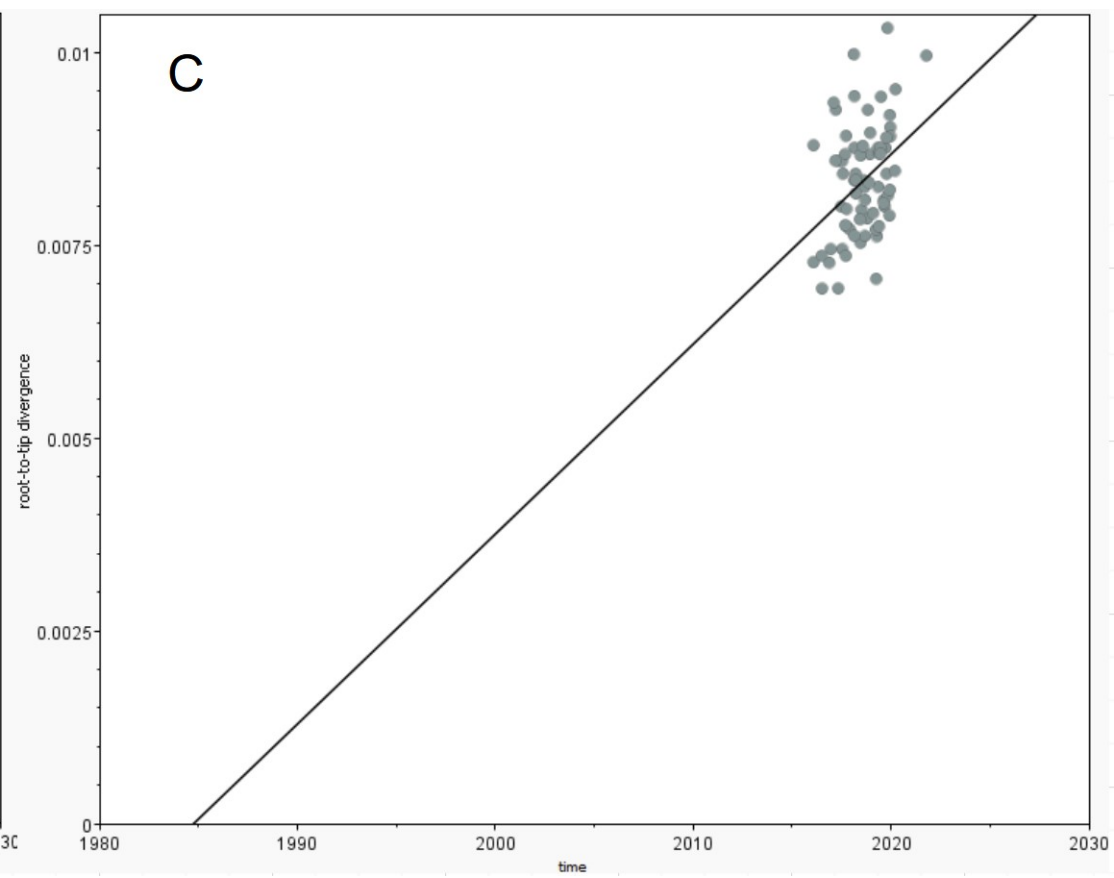

Supplement: veae114_Supp [file veae114_supp.zip › suppl_data/S Figure 5. Tempest root-to-tip (RTT) regression plot.pdf]

# Average Markov Jump Counts

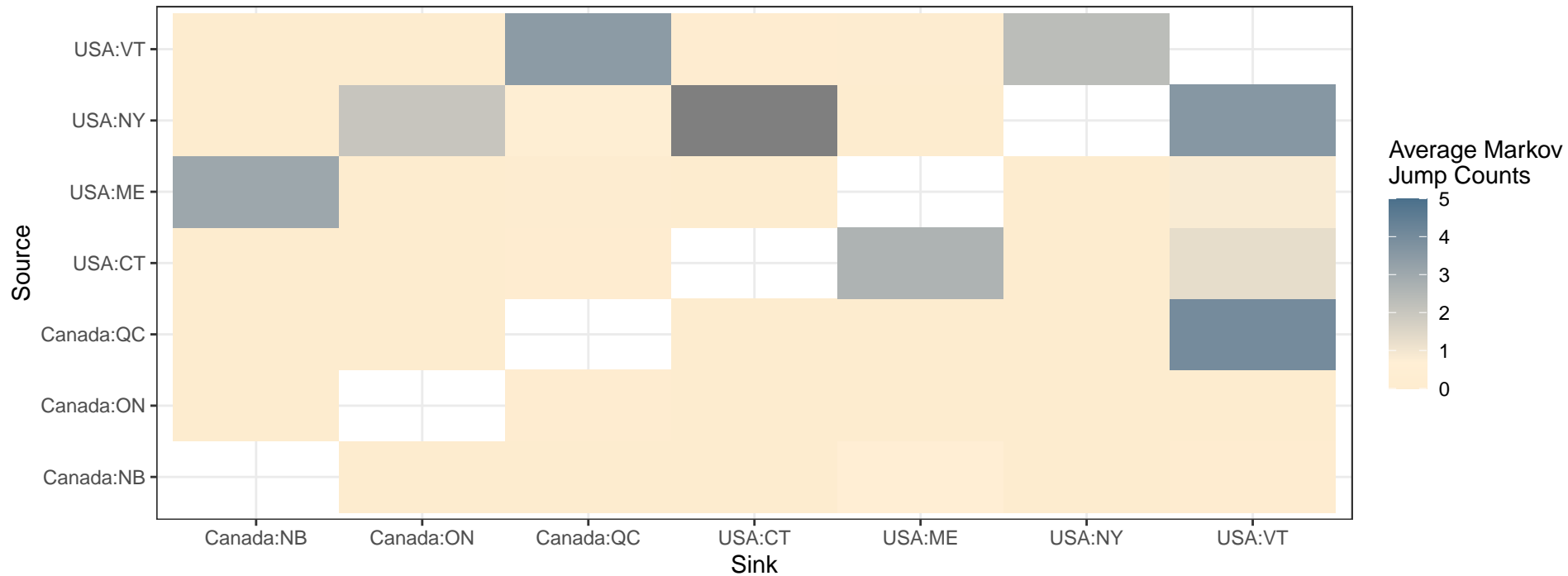

Supplement: veae114_Supp [file veae114_supp.zip › suppl_data/S Figure 6. Average Markov jump counts, North America.pdf]

# Average Markov Jump Counts

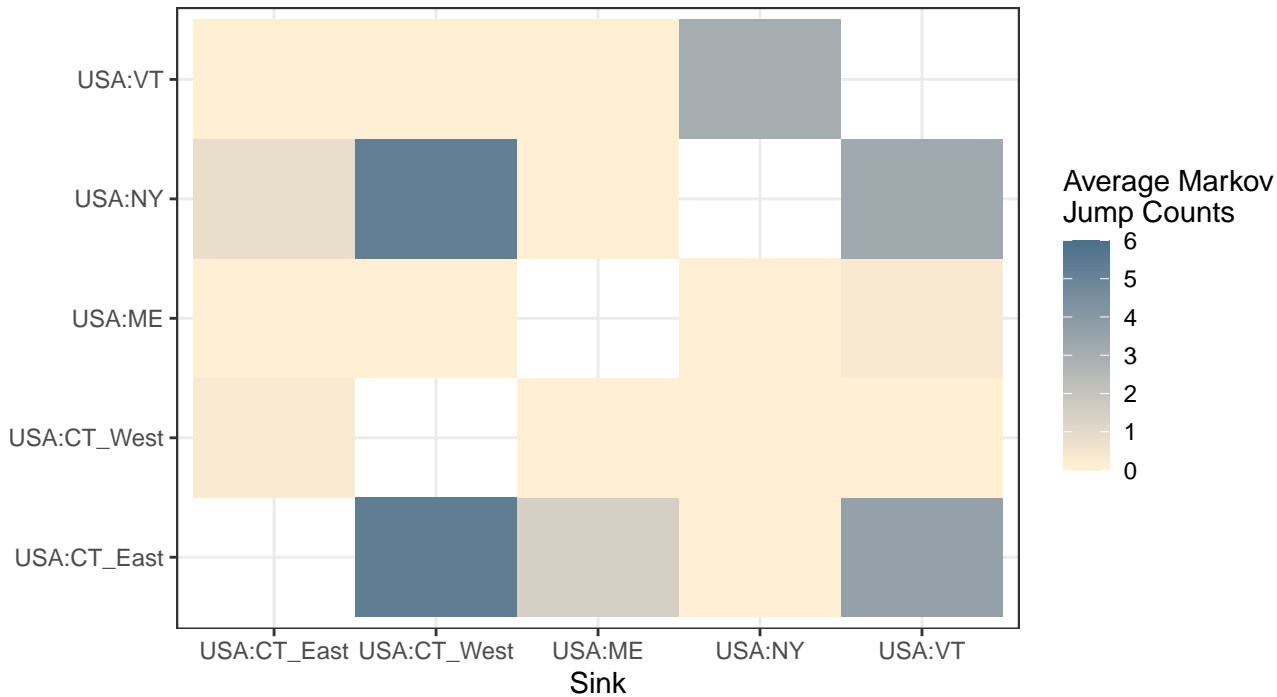

Supplement: veae114_Supp [file veae114_supp.zip › suppl_data/S Figure 7. Average Markov jumps, Connecticut River.pdf]

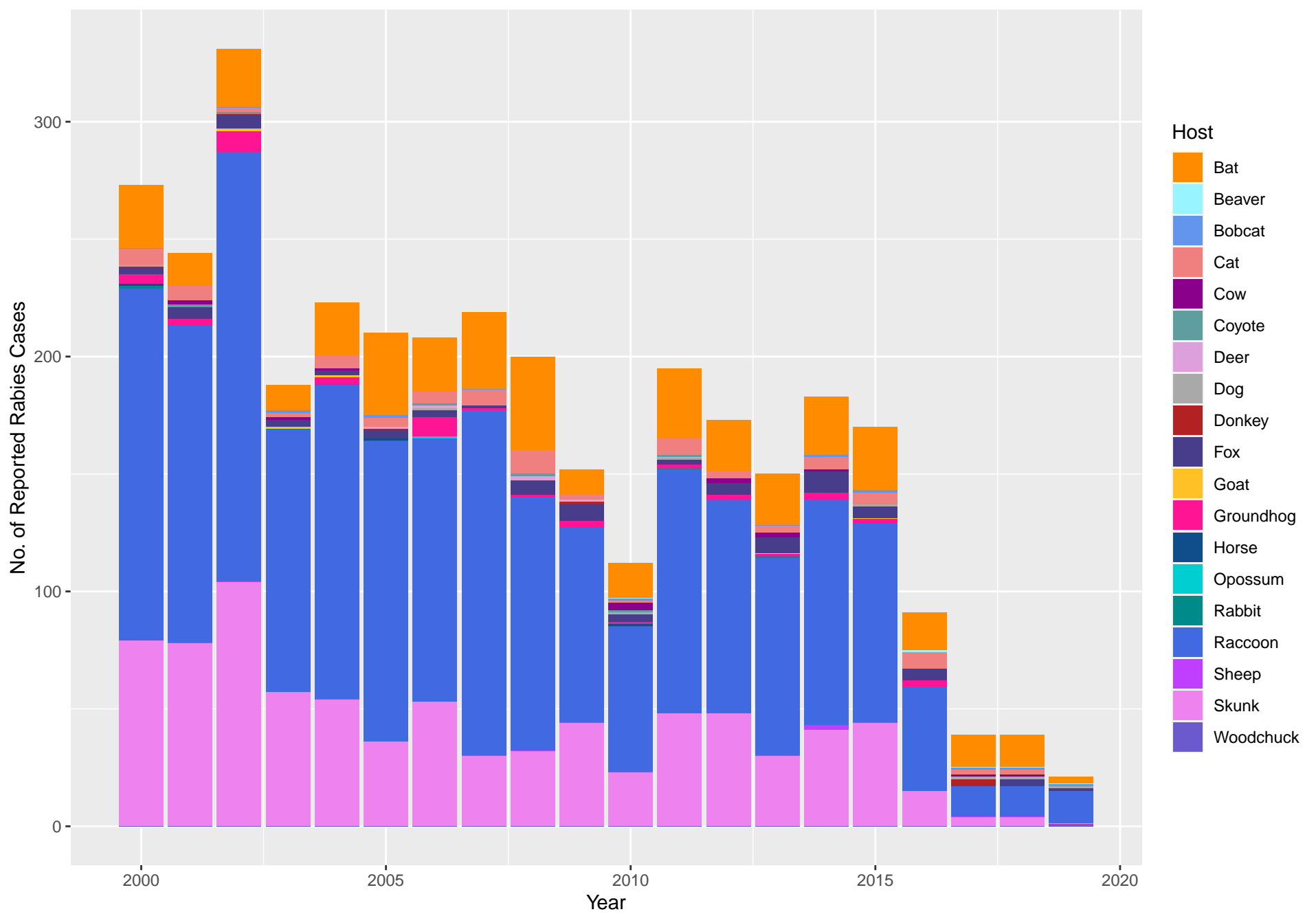

Supplement: veae114_Supp [file veae114_supp.zip › suppl_data/S Figure 8. Reported Rabies Virus Cases in Connecticut, by Species, 2000-2019.pdf]

S1

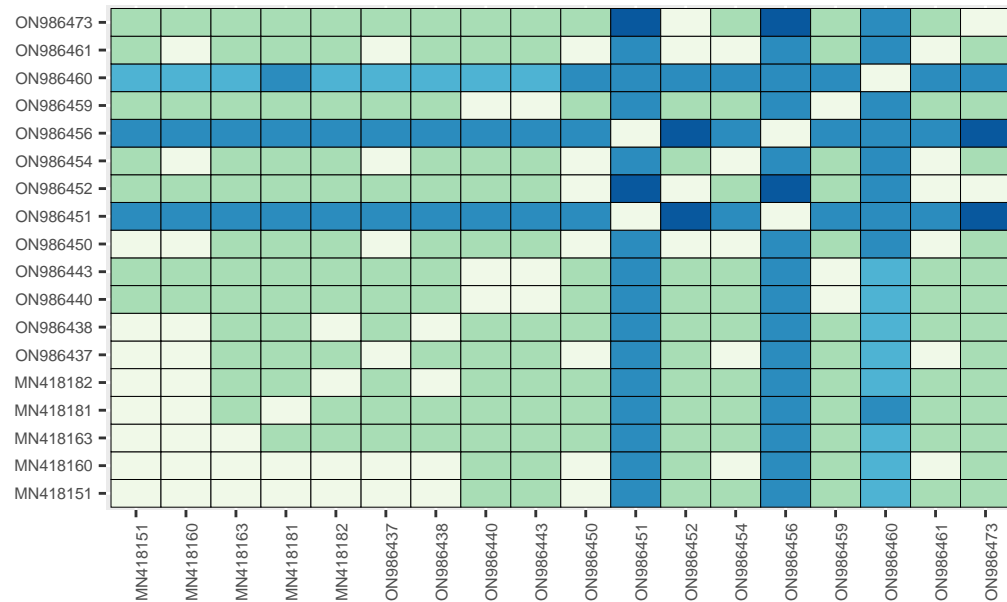

S2

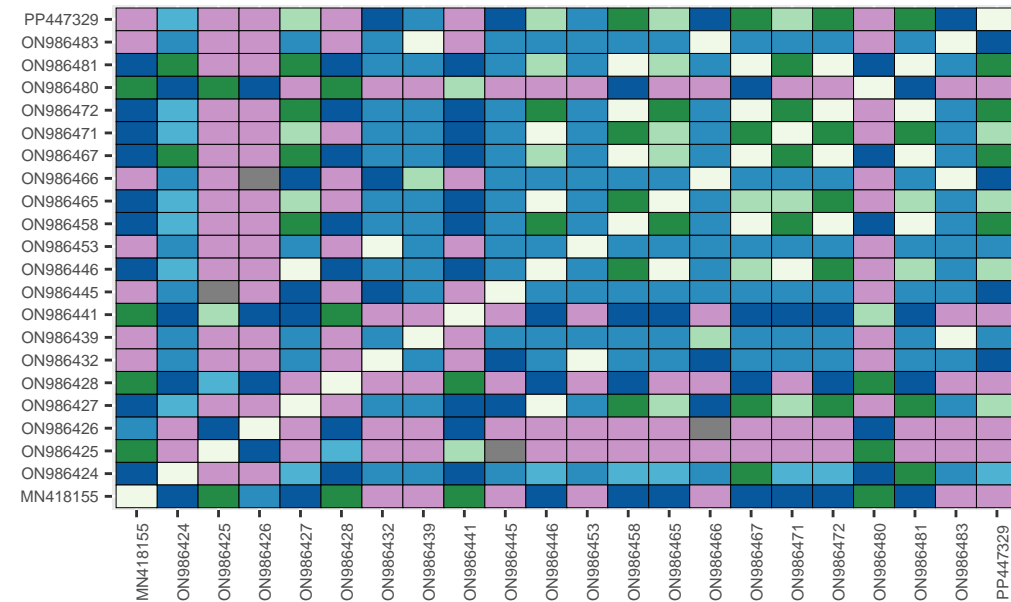

S3

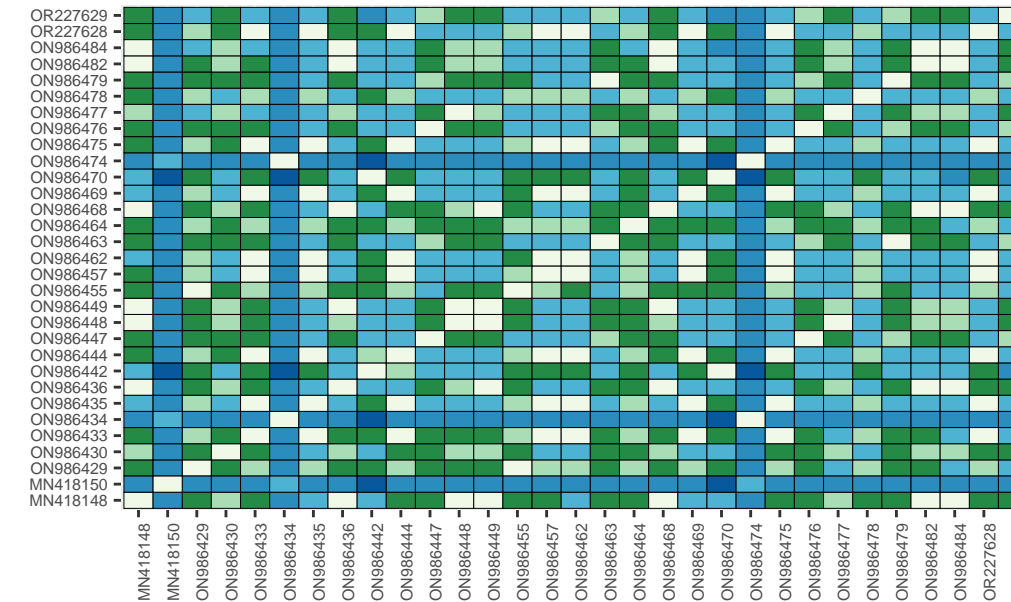

S1/S2

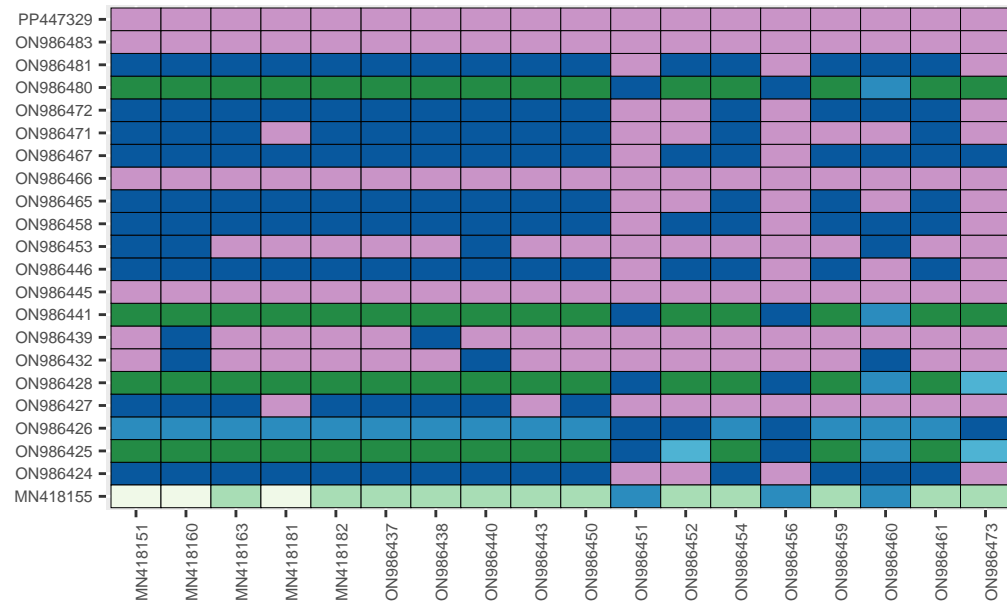

S2/3

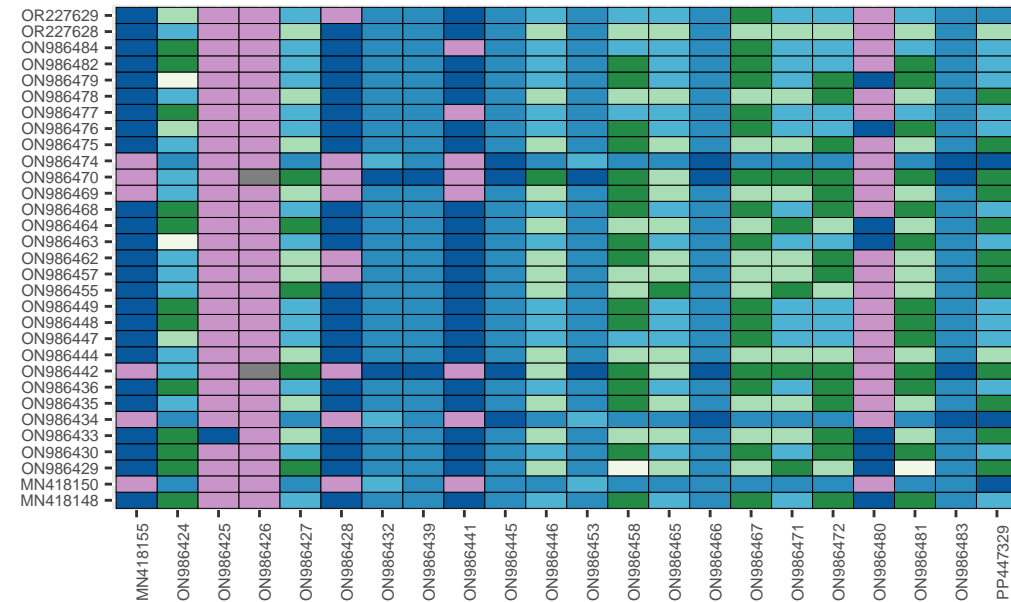

S1/3

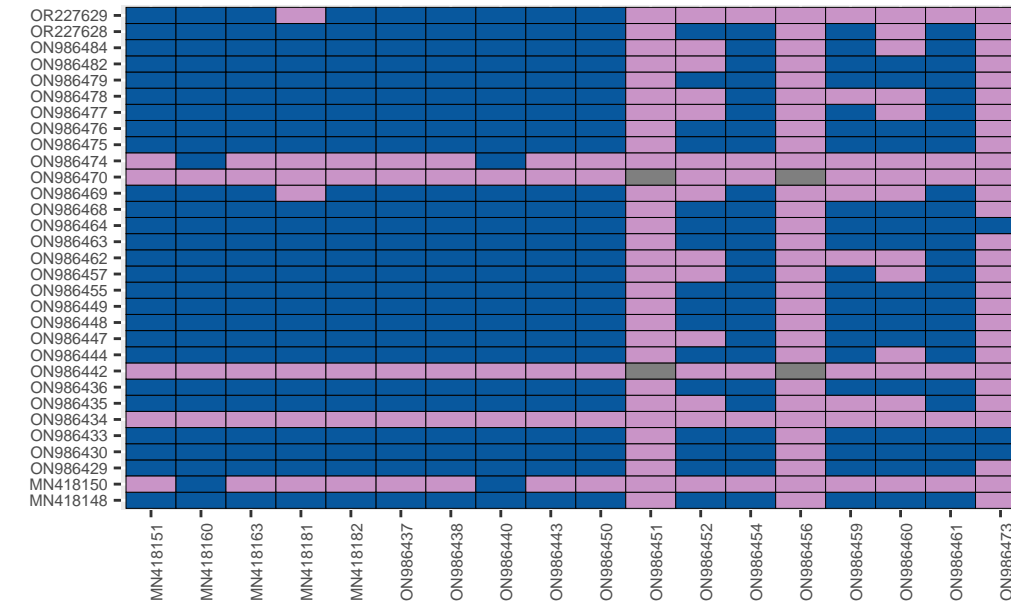

Supplement: veae114_Supp [file veae114_supp.zip › suppl_data/S Figure 9. RRV pairwise genetic distance between isolates, Section.pdf]
